# Genomewide Study of Epigenetic Biomarkers of Opioid Dependence in European- American Women

**DOI:** 10.1038/s41598-019-41110-7

**Published:** 2019-03-15

**Authors:** Janitza L. Montalvo-Ortiz, Zhongshan Cheng, Henry R. Kranzler, Huiping Zhang, Joel Gelernter

**Affiliations:** 1 Division of Human Genetics, Department of Psychiatry, Yale University School of Medicine, New Haven, CT USA; 2 VA CT Healthcare Center, West Haven, CT USA; 3 University of Pennsylvania Perelman School of Medicine, Department of Psychiatry, Center for Studies of Addiction and Crescenz Veterans Affairs Medical Center, Philadelphia, PA USA; 4 Departments of Psychiatry and Medicine (Biomedical Genetics), Boston University School of Medicine, Boston, MA USA; 5 Departments of Genetics and Neuroscience, Yale University School of Medicine, New Haven, USA

**Keywords:** DNA methylation, Epigenetics and behaviour

## Abstract

There is currently an epidemic of opioid use, overdose, and dependence in the United States. Although opioid dependence (OD) is more prevalent in men, opioid relapse and fatal opioid overdoses have recently increased at a higher rate among women. Epigenetic mechanisms have been implicated in the etiology of OD, though most studies to date have used candidate gene approaches. We conducted the first epigenome-wide association study (EWAS) of OD in a sample of 220 European-American (EA) women (140 OD cases, 80 opioid-exposed controls). DNA was derived from whole blood samples and EWAS was implemented using the Illumina Infinium HumanMethylationEPIC array. To identify differentially methylated CpG sites, we performed an association analysis adjusting for age, estimates of cell proportions, smoking status, and the first three principal components to correct for population stratification. After correction for multiple testing, association analysis identified three genome-wide significant differentially methylated CpG sites mapping to the *PARG*, *RERE*, and *CFAP77* genes. These genes are involved in chromatin remodeling, DNA binding, cell survival, and cell projection. Previous genome-wide association studies have identified *RERE* risk variants in association with psychiatric disorders and educational attainment. DNA methylation age in the peripheral blood did not differ between OD subjects and opioid-exposed controls. Our findings implicate epigenetic mechanisms in OD and, if replicated, identify possible novel peripheral biomarkers of OD that could inform the prevention and treatment of the disorder.

## Introduction

The lifetime U.S. prevalence of opioid dependence (OD) is 0.3% and of opioid abuse, 1.1%^1^. These result in an estimated annual cost of $78.5 billion^2^. Further, opioids are the single greatest cause of accidental fatal overdose in the United States^3–6^, which until recently was mainly caused by heroin and prescription opioid pain relievers. However, recent data show fentanyl emerging as a major problem^7^. In the United States, OD and opioid abuse have become an epidemic^8^ and fatal opioid overdoses have quadrupled in recent years^9^.

Genome-wide association studies (GWAS) have been widely used to identify genetic factors that predispose to drug dependence^10^. GWASs from our lab have identified risk variants for OD; the most compelling implicated potassium and calcium signaling^11^, and more recently, *RGMA*, the gene that encodes repulsive guidance molecule A, a central nervous system axon guidance protein^12^. However, because these associations account for only a small proportion of the known heritability, additional mechanisms are certainly involved in the risk for OD.

Epigenetic mechanisms, which underlie some of the interplay between environmental factors and genes, are implicated in drug abuse risk. Several studies have shown that DNA methylation, the most studied epigenetic mechanism in humans, is altered by opioid abuse or dependence. Overall methylation in peripheral blood DNA is greater in OD subjects than controls^13^. Candidate epigenetic studies at the *OPRM1* gene have shown that increased DNA methylation at this locus is associated with OD, based on whole blood cells^13–16^ and brain tissue^17^. A study from our lab examining 16 CpGs in the *OPRM1* promoter region showed that three closely mapped CpGs within the promoter are hypermethylated in cases with comorbid AD and OD compared to controls^18^.

However, these studies have yielded inconsistent findings^19^ and have mostly been performed in men. Women are 48% more likely than men to use prescription drugs and to be prescribed opioids, so it is particularly important for studies of these traits to include females. Further, very few studies to date have examined genome-wide DNA methylation changes associated with substance dependence and no methylation studies have evaluated OD specifically. There has been one small (*n* = 48) genome-wide DNA methylation study of methadone dose^20^.

We examined genome-wide DNA methylation changes associated with OD in a population of European-American (EA) women to identify peripheral biomarkers that can inform preventive and treatment strategies for the disorder.

## Materials and Methods

### Sample

The study sample consisted of 220 EA women (mean age = 40 ± 13.5 years) recruited at two clinical sites: Yale University School of Medicine (APT Foundation, New Haven, CT), and the University of Connecticut Health Center (Farmington, CT). The participants were selected from a sample of approximately 13,000 individuals recruited in the course of our NIH-funded studies of the genetics of alcohol and drug dependence^11,21–23^. The study was approved by the Yale Humans Investigation Committee, and research was performed following their guidelines. All subjects provided written informed consent, and certificates of confidentiality were obtained from the National Institute on Drug Abuse (NIDA) and the National Institute on Alcohol Abuse and Alcoholism (NIAAA).

Subjects were interviewed using the Semi-Structured Assessment for Drug Dependence and Alcoholism (SSADDA), a polydiagnostic assessment for psychiatric traits used to derive a diagnosis of DSM-IV OD^12^. All subjects were opioid exposed (based on self-reported lifetime opioid use >10 times). The sample consisted of 140 OD cases and 80 opioid-exposed controls. Demographic and clinical characteristics of the sample are shown in Table 1. Polydrug use, that is, the prevalence of more than one substance dependence diagnosis, is present in 74% of the sample; 95.7% in OD cases and 36.3% in opioid-exposed controls. AD is present in 41.8% and cocaine dependence (CocD) is present in 61.8% of the sample. Among opioid-exposed controls, 23.7% are AD subjects and 31.3% are CoCD subjects. Among OD cases, 52.1% are AD subjects, and 79.3% are CoCD subjects.

**Table 1:** Demographic and Clinical Characteristics of the Study Cohort.

	Total *N* = 220
**Age**	
	40.0 ± 13.5
In opioid-exposed controls	46.0 ± 14.6
In OD cases	35.0 ± 9.4
Smoking status	
% Current smoker	70.50%
In opioid-exposed controls, % current smoker	33.80%
In OD cases, % current smoker	91.40%
Polydrug use	
% Polydrug use	74.00%
In opioid-exposed controls, % polydrug use	95.70%
In OD cases, % polydrug use	36.30%
Opioid dependence	
% OD cases	63.60%
Alcohol dependence	
% AD cases	41.80%
In opioid-exposed controls, % AD cases	23.70%
In OD cases, % AD cases	52.10%
Cocaine dependence	
% CocD cases	61.80%
In opioid-exposed controls, %CocD cases	31.30%
In OD cases, % CocD cases	79.30%

Abbreviations: OD = Opioid dependence; AD = Alcohol dependence; CocD = Cocaine dependence.

### Genomic DNA extraction, Bisulfite Modification, Methylation Array

Genomic DNA (500 ng) was extracted from whole blood using PAXgene Blood DNA kits (Qiagen, Valencia, CA, USA) and standard procedures. Genomic DNA (500 ng) was treated with bisulfite reagents using a EZ-96 DNA methylation kit (Zymo Research, Orange, CA, USA) according to the manufacturer’s protocol. Bisulfite-converted DNA samples were used in the array-based genome-wide DNA methylation assay. Methylation status was assessed using the Illumina Infinium Human MethylationEPIC BeadChip (Illumina, San Diego, CA, USA), which interrogates DNA methylation >800,000 loci across the genome at single-nucleotide resolution.

### Quality Control and Normalization

Genome-wide DNA methylation assays were conducted at the Yale Center for Genome Analysis. GenomeStudio software (Illumina) was used to generate β values for each CpG site; β values were defined as *M*/(*M* + *U* + α), where *M* is the total methylated signal and *U* the total unmethylated signal, ranging from 0.0 to 1.0, with α = 100 added to stabilize beta values when both *M* and *U* are small.

Quality control was performed based on a pipeline using the ‘minfi’ R package (Bioconductor 1.8.9)^24^. CpG sites with detection p-value > 0.001 were removed to ensure that only high-confidence probes were included. Probes with annotated single nucleotide polymorphisms (SNPs) at single-base extension (SBE/CPG) sites (via the Single Nucleotide Polymorphism database 137 [National Center for Biotechnology Information]) or mapped to multiple places in the genome or to sex chromosomes were also excluded. Combat method in the ‘sva’ package^25^ was applied to correct for batch effects associated with sample plate and cohort group. Functional normalization was conducted using the “Preprocessfunnorm” function in the ‘minfi’ R package^24^, which uses internal control probes present on the array to control for between-array technical variation, thereby outperforming other approaches^26^. Density plots were generated to evaluate the distribution of beta (β) values before and after functional normalization (Supplementary Fig. 1). After quality control and normalization, a total of 790,677 CpG sites (91% of possible sites) were left for subsequent analysis.

Because methylation values at CpG sites can be cell-type specific^27^, we conducted a cell composition analysis following a published method^28^. The relative proportion of each cell type in our heterogenous peripheral blood samples was estimated by implementing the ‘minfi’ function “estimateCellCounts” from the R package ‘Flow-Sorted.Blood.450k’^24^.

To adjust for possible population stratification within the EA subjects, a methylation-based principal component (PC) approach was conducted based on sets of CpG sites within 50 kb of SNPs using the 1000 Genomes Project variants with minor allele frequency (MAF) > 0.1^29^.

### Statistical analysis

All statistical analyses were performed within R 3.4.0 (www.r-project.org). To identify differentially methylated CpG sites associated with OD, EWAS was conducted using the ‘cpg.assoc’ function from the ‘minfi’ R package^30^, adjusting for age, estimated cell proportions (i.e., CD8T, CD4T, NK, C cells, monocytes and granulocytes), and the first three population stratification PCs. Given the broad impact of smoking on DNA methylation across the genome^31^ and the comorbidity of OD with smoking behavior, we also adjusted for smoking status. We used Bonferroni correction to adjust for multiple testing (*p* value for significance set at 5.9 × 10^−8^).

### Methylation Quantitative Trait Loci Analysis

Methylation quantitative trait loci (meQTL) analysis was conducted to examine whether methylation patterns at GWS CpG sites interacted with genotype variation. Genotype data were available for 141 subjects included in the EWAS analysis. Genotype information, imputation, and quality control information is provided in our recent OD GWAS^12^. SNPs within 1 MB of the CpG site (hg19 reference genome) were included in the meQTL analysis. We conducted a linear regression analysis using PLINK 1.9^32^ adjusting for the same covariates as in the EWAS. Analyses were performed separately based on the genotyping array (these included the HumanOmni1-Quad v1.0 and the HumanCore Exome array, both from Illumina, Inc., San Diego, CA) and then combined by meta-analysis using the inverse variance method implemented in PLINK 1.9. meQTL analysis were also conducted in OD cases (*n* = 121) and opioid-exposed controls (*n* = 29), separately.

### Estimated Epigenetic Age

Methods are described in the Supplementary section.

## Results

### Epigenome-wide association analysis

**Figure 1:**
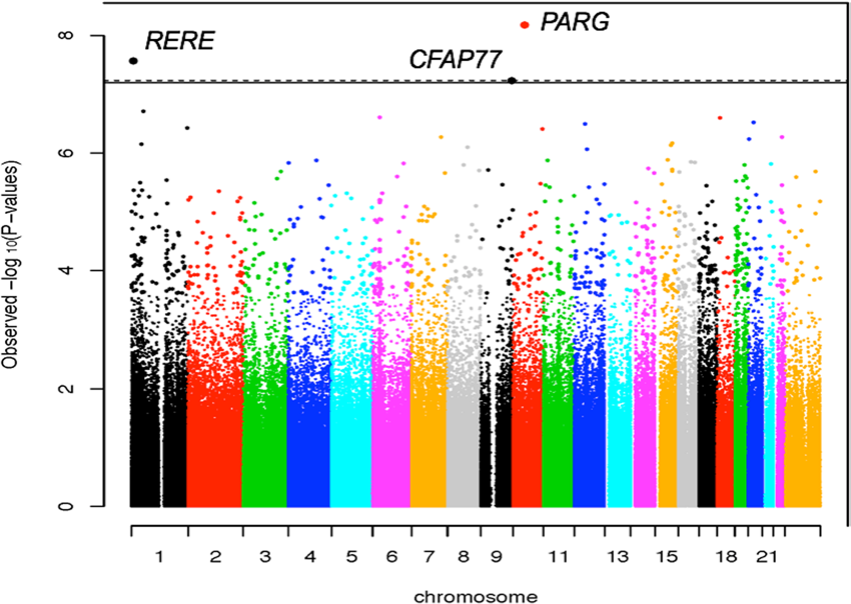
Manhattan plot. The Manhattan plot depicts the association between DNA methylation and opioid dependence (OD) in European American (EA) women (*n* = 220). The horizontal dotted line represents the genome-wide significant (GWS) threshold of *p* < 5.9 × 10^−8^.

Three CpG sites were genome-wide significant (GWS) after Bonferroni correction (p < 5.9 × 10^−8^). Table 2 lists the top 10 differentially methylated CpG sites associated with OD in EA women. A Manhattan plot is shown in Fig. 1. Supplementary Figure 2 illustrates the quantile-quantile (QQ) plot for the *p*-values of the association between DNA methylation and OD. There was no evidence of inflation (λ = 1.00).

**Table 2:** Top 10 Differentially Methylated CpG Sites Associated with OD in EA women (Bonferroni-corrected p-value significance threshold, *p* < 5.9 × 10^−8^).

IlmnID	UCSC gene symbol	UCSC gene name	Chr	Position	CpG Position	F statistic	*P* value
**cg17426237**	***PARG***	**Poly(ADP-ribose) glycohydrolase**	**10**	**51463156**		**36.70**	**6.77E-09**
**cg21381136**	***RERE***	**Arginine-glutamic acid dipeptide repeats**	**1**	**8445818**	**5′UTR; Gene Body**	**33.48**	**2.75E-08**
**cg18177613**	***CFAP77***	**Cilia and flagella associated protein 77**	**9**	**135359969**	**Gene Body**	**31.76**	**5.89E-08**
cg23095642	*OSBPL9*	Oxysterol binding protein-like 9	1	52081938	TSS1500	29.05	1.97E-07
cg04983519	*EHMT2*	Euchromatic histone-lysine N-methyltransferase 2	6	31853647	Gene Body	28.52	2.51E-07
cg24210426	*[PTPN2]*	Protein tyrosine phosphatase, non-receptor type 2	18	12780803		28.47	2.56E-07
cg20960678	*[SYNDIG1]*	Synapse differentiation inducing 1	20	24659877		28.10	3.04E-07
cg11395372	*TUBA1C*	Tubulin alpha 1c	12	49641058	5′UTR; Gene body	27.95	3.25E-07
cg05157272	*[CNST]*	Connexin sorting protein	1	246862256		27.61	3.79E-07
cg04379122	*[LINC01163]*	Long intergenic non-protein coding RNA 1163	10	130119293		27.54	3.92E-07

**Figure 2:**
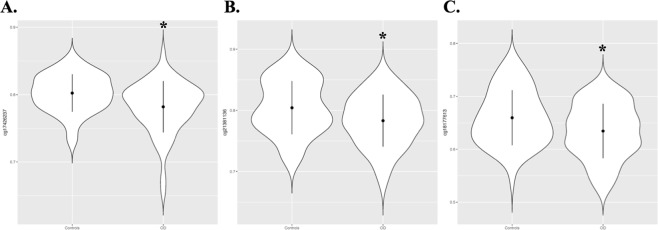
Violin plots of genome-wide significant (GWS) CpG sites associated with opioid dependence (OD). DNA methylation levels (beta values) of GWS CpG sites associated with OD are shown: (**A**) cg17426237 (*PARG* gene), (**B**) cg21381136 (*RERE* gene), and (**C**) cg18177613 (*CFAP77* gene) in opioid-exposed controls and OD cases. All CpG sites showed hypomethylation associated with OD. ^*^Represents GWS threshold of *p* < 5.9 × 10^−8^.

All three of the GWS differentially methylated CpG sites showed decreased DNA methylation in OD subjects (Fig. 2). The top GWS CpG site identified was cg17426237 (chr 10:51463156, *p* = 6.77 × 10^−9^) located within the poly(ADP-ribose) glycohydrolase (*PARG*) gene. The other two GWS differentially methylated CpG sites were cg21381136 (*RERE*; “arginine-glutamic acid dipeptide repeats”; chr1:8445818; *p* = 2.75 × 10^−8^), and cg18177613 (*CFAP77*; “cilia and flagella associated protein 77”; chr9:135359969; *p* = 5.89 × 10^−8^). Functional annotation of the GWS CpGs showed that two sites were located in the gene body (cg21381136 and cg18177613) and one in the 5′UTR region (cg21381136). Further, cg21381136 is an enhancer and cg18177613 has three DNase hypersensitivity sites, an indicator of open chromatin. DNA methylation patterns in whole blood and multiple brain regions for cg17426237 are shown in Supplementary Fig. 3. Gene expression patterns in blood and multiple brain tissues for *PARG* and *RERE* are shown in Supplementary Fig. 4.

Three other differentially methylated CpG sites were near GWS: cg23095642 located at *OSBPL9* (“Oxysterol binding protein-like 9”), cg04983519 at *EHMT2* (“euchromatin histone-lysine N-methyltransferase 2”), and cg11395372 at *TUBA1C* (“tubulin alpha 1c”) (Table 2).

### Sensitivity analysis

OD subjects have a higher rate of other substance use disorders, including AD and CoCD, than controls. To account for the differences in AD and CocD prevalence between cases and controls, we included these two diagnoses as covariates along with age, cell type proportion, the first three PCs and smoking status for the GWS CpG sites. After adjusting for AD and CocD, associations of *PARG*, *RERE*, and *CFAP77* CpG sites with OD slightly decreased (*p* = 8.01 × 10^−9^, 3.69 × 10^−8^, and 3.45 × 10^−7^, respectively). The magnitudes of the effects of these variables, as well as current smoking, are shown in Supplementary Fig. 5.

**Figure 3:**
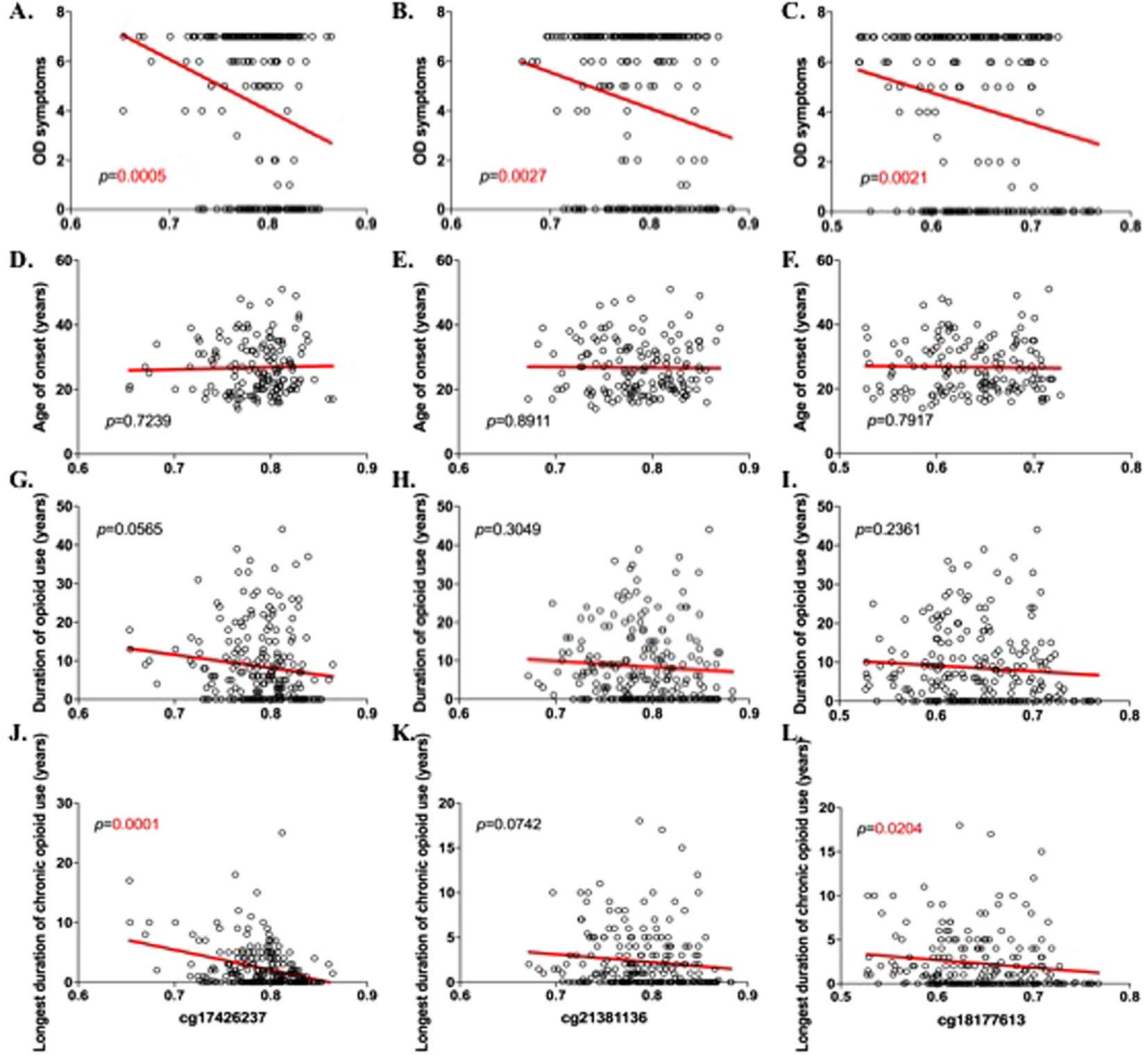
Association between genotype data at rs2611513 and DNA methylation levels at GWS CpG site cg17426237. rs2611513 associated with DNA methylation levels at cg17426237; C allele associated with lower DNA methylation in (**A**) all sample (*n* = 141, *p* = 0.025), and (**B**) OD cases (*n* = 112; *p* = 0.042). No association was obtained in (**C**) opioid-exposed controls (*n* = 29; NS). ^*^Represents *p* < 0.05.

DNA methylation levels at these CpG sites were also associated with OD related traits such as OD symptom count (cg17426237, *p* = 0.0005; cg21381136, *p* = 0.0027; cg18177613, *p* = 0.0021; Fig. 3A–C), and longest duration of chronic opioid use (cg17426237, *p* = 0.0001; cg18177613, *p* = 0.0204; Fig. 3J,L). A trend-level possible association was also observed for the duration of opioid use (cg17426237, *p* = 0.0565; Fig. 3G), and longest duration of chronic opioid use (cg21381136, *p* = 0.0742; Fig. 3K). No association was observed with OD age of onset. Significant associations between DNA methylation at these CpG sites and OD-related traits are in the same direction as with OD: DNA hypomethylation at these CpG sites is associated with OD. In this dataset, OD status correlates with OD symptoms (*n* = 218, 2 missing values, *p* < 0.0001), duration of opioid use (*n* = 217, 3 missing values, *p* < 0.0001), and longest duration of chronic opioid use (*n* = 218, 2 missing values, *p* < 0.0001), but not with OD age of onset (*n* = 120, OD cases only, *p* = 0.9048).

### meQTL analysis

**Figure 4:**
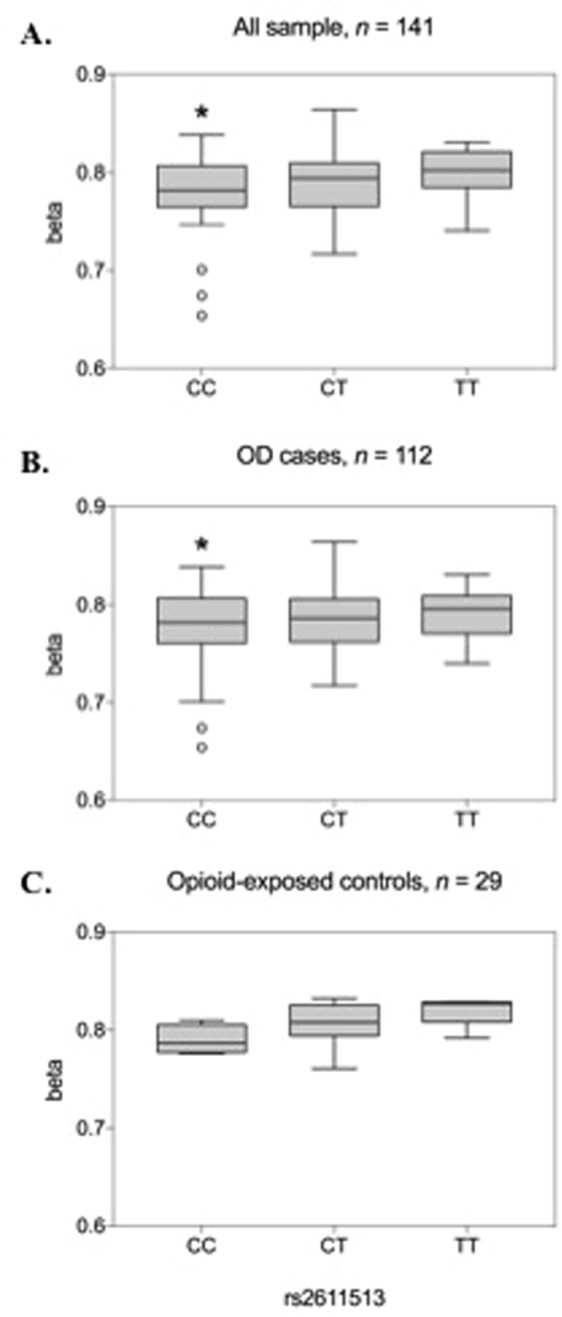
Association between genome-wide significant (GWS) CpG sites and opioid dependence (OD)-related traits. DNA methylation (beta values) of GWS CpG sites associated with OD-related traits are shown: (**A**–**C)** OD symptoms, (**D**–**F)** age of onset (years), (**G**–**I**) duration of opioid use (years), (**J**–**L)** longest duration of chronic opioid use (years). Significant threshold is set at *p* < 0.05.

After meta-analysis, a SNP (rs2611513) mapped to *PRKG1* was nominally significant in association with methylation patterns of cg17426237 at *PARG* (*n* = 141, *p* = 0.025; Fig. 4A). This SNP was nominally significant (β = 0.17, *p* = 0.02) in our most recent EA OD GWAS^12^. The risk allele (C) for OD at rs2611513^33^ was associated with decreased methylation at cg17426237. In the OD EWAS, cg17426237 also showed lower methylation. To determine whether the SNP is also a meQTL in OD cases and opioid-exposed controls considered separately, we further correlated the genotype of rs2611513 with cg17426237 in subset samples of cases and controls. We found that rs2611513 genotype was nominally significant in association with methylation patterns of cg17426237 at *PARG* gene in OD cases (*n* = 121, *p* = 0.042; Fig. 4B). In opioid-exposed controls, no association was observed, possibly due to limited power from the small sample size (*n* = 29; Fig. 4C).

### Estimated epigenetic age in the peripheral blood of OD subjects and opioid-exposed controls

As expected, chronological age was significantly correlated with methylation age (DNAm Age) (*r* = 0.93, *p* < 0.0001) in the full sample of 220 subjects (Supplementary Fig. 6A). No significant differences were observed in the age acceleration residual between OD subjects and opioid-exposed controls (Supplementary Fig. 6B).

## Discussion

This is the first epigenome-wide association study of OD. After correction for multiple testing, we identified three GWS CpG sites in EA women, which map to *PARG*, *RERE*, and *CFAP77 –* genes implicated in chromatin remodeling, DNA binding, cell survival, and cell projection. Among these, one CpG site showed a significant association with a *cis-*genetic variant. The results support the interpretation that DNA methylation differences could be attributable to both environmental and genetic influences.

Our top GWS CpG site, cg17426237, is located in the *PARG* gene. The protein product of this gene is a catabolic enzyme of poly (ADP-ribose), involved in various cellular processes including DNA repair, transcription, and the modulation of chromatic structure. Methylation of this CpG site also occurs in brain tissue across multiple brain regions (Supplementary Fig. 3). *PARG* gene is highly expressed in human brain (Supplementary Fig. 4A), with its highest expression in cerebellar tissue.

The second top GWS CpG site identified, cg21381136, maps to the *RERE* gene, which is also highly expressed in brain (Supplementary Fig. 4B). SNPs at this locus have been identified in GWASs of schizophrenia^33–35^ and in the cross-disorders analysis from the Psychiatric Genomics Consortium, which included autism spectrum disorder, attention deficit-hyperactivity disorder, bipolar disorder, major depressive disorder^33^; and educational attainment^36^. The encoded protein (RERE) is involved in transcriptional repression during embryonic development, chromatin remodeling, and cell survival. RERE interacts with EHMT2^37^, also known as G9A, a histone methyltransferase involved in transcriptional repression. A CpG site at *EHMT2*, cg04983519, is among the top 10 differentially methylated CpG sites associated with OD identified herein. Considered together, these results implicate the gene regulation pathway.

A third GWS CpG site that we identified, cg18177613, is located at the *CFAP77* gene, involved in cell projection and protein binding. The association of the GWS CpG sites identified with OD appears to be mostly independent of comorbid AD and CocD in OD subjects compared to opioid-exposed controls. Further, DNA methylation at these CpG sites is also associated with OD-related traits such as OD symptom count and longest duration of chronic opioid use in the same direction as with OD. Additional differentially methylated CpG sites with suggestive associations mapped to genes implicated in metabolism (*OSBPL9*), transcriptional regulation (*EHMT2*), and protein binding and axon guidance (*TUBA1C*).

When we examined methylation patterns of these GWS CpG sites in relation to genotype, we determined that the methylation state of cg17426237 (*PARG*) was associated with rs2611513, which maps to *PRKG1*. Rs2611513 was nominally significantly associated to OD (β = 0.17, *p* = 0.02) in EAs in our published GWAS^12^; the OD risk allele is associated with decreased methylation at this CpG site. This shows the same effect direction as in the current EWAS analysis, where OD subjects show decreased methylation at this CpG site, and the risk variant predicted decreased methylation. These findings suggest that risk variants could reflect epigenetic regulatory loops, modulating gene expression by an epigenetic mechanism.

When examining the estimated methylation age in the peripheral blood, no differences were observed between opioid-exposed controls and OD subjects. In a previous study conducted in human postmortem striatal tissue, neurons of heroin abusers exhibited a younger epigenetic age than control subjects^38^. The differences observed could be due to cell type specificity in DNA methylation age associated with opioid use. Another possibility is differences in the sample composition between the studies; for example, control subjects for whom exposure to opioids were not required, compared to the current study, in which all controls were opioid-exposed. However, larger samples are needed to investigate this further.

This study has several strengths. It is the largest EWAS sample to date for the study of OD. Important confounding variables were considered in the EWAS analysis, such as smoking status, population stratification, and cell type composition. The sample size is moderate, but power was increased by using a carefully ascertained sample of a single ancestry and sex for both cases and opioid-exposed controls. Further, we integrated genetic and epigenetic information to investigate the interplay between genetic and environmental mechanisms in OD.

Our findings should be interpreted in the context of several limitations. Identifying a validation data set was not possible at this point because this is, to our knowledge, the first EWAS study of OD. Thus, future studies are needed to examine whether the GWS CpG sites identified can be replicated in an independent sample, in males, or in other populations. Another limitation is the use of peripheral tissue to investigate DNA methylation differences associated with OD. We used different publicly available datasets to conduct a cross-tissue proxy of the methylation and gene expression patterns of the genes identified. Further, the purpose of this study was to identify potential peripheral biomarkers of OD that could inform prognosis and potential treatments in humans. Although brain is a tissue of greater interest, it is inaccessible in living subjects.

Given the cross-sectional design of the study, we were unable to determine whether the changes in DNA methylation caused or were a consequence of OD. Future longitudinal studies are needed to investigate this further. Larger samples will allow for better power to investigate the effect of additional confounding factors such as polydrug use (present in 74% of the sample; 95.7% in OD cases and 36.3% in opioid-exposed controls) or comorbidity with psychiatric diagnoses. Improved power will also likely facilitate the identification of additional novel loci. Despite these limitations, this is the first study of its kind to identify DNA methylation signatures associated with OD. Based on our sensitivity analysis in our top signals, the association between the GWS CpG sites identified and OD goes beyond the influence of comorbidity with other drug dependencies (i.e., AD and CoCD). These data suggest that hypomethylation at these loci may, if replicated, be used as specific biomarkers for OD.

## Conclusions

This study is the first genome-wide DNA methylation association study of OD in women. After correction for multiple testing, we identified three GWS DNA methylation sites in genes involved in chromatin remodeling, DNA binding, and cell processes. Further, genetic variants in one of these genes have previously been identified in GWASs of psychiatric disorders.

## Supplementary information

Supplementary Information

## References

[1] Compton WM, Volkow ND (2006). Major increases in opioid analgesic abuse in the United States: concerns and strategies. Drug and alcohol dependence..

[2] Florence CS, Zhou C, Luo F, Xu L (2016). The Economic Burden of Prescription Opioid Overdose, Abuse, and Dependence in the United States, 2013. Medical care..

[3] Compton, W. M. & Volkow, N. D. Abuse of prescription drugs and the risk of addiction. *Drug and alcohol dependence*. S4–7 (2006). 10.1016/j.drugalcdep.2005.10.020 16563663

[4] Paulozzi LJ (2006). Opioid analgesic involvement in drug abuse deaths in American metropolitan areas. American journal of public health..

[5] Paulozzi LJ, Budnitz DS, Xi Y (2006). Increasing deaths from opioid analgesics in the United States. Pharmacoepidemiology and drug safety..

[6] Paulozzi LJ, Xi Y (2008). Recent changes in drug poisoning mortality in the United States by urban-rural status and by drug type. Pharmacoepidemiology and drug safety..

[7] Mercado MC (2018). Increase in Drug Overdose Deaths Involving Fentanyl-Rhode Island, January 2012-March 2014. Pain medicine..

[8] Okie S (2010). A flood of opioids, a rising tide of deaths. The New England journal of medicine..

[9] Volkow ND, Frieden TR, Hyde PS, Cha SS (2014). Medication-assisted therapies–tackling the opioid-overdose epidemic. The New England journal of medicine..

[10] Gelernter J (2015). Genetics of complex traits in psychiatry. Biological psychiatry..

[11] Gelernter J (2014). Genome-wide association study of opioid dependence: multiple associations mapped to calcium and potassium pathways. Biological psychiatry..

[12] Cheng, Z. *et al*. Genome-wide Association Study Identifies a Regulatory Variant of RGMA Associated With Opioid Dependence in European Americans. *Biological psychiatry* (2018). 10.1016/j.biopsych.2017.12.016 PMC6041180 29478698

[13] Chorbov VM, Todorov AA, Lynskey MT, Cicero TJ (2011). Elevated levels of DNA methylation at the OPRM1 promoter in blood and sperm from male opioid addicts. Journal of opioid management..

[14] Nielsen DA (2009). Increased OPRM1 DNA methylation in lymphocytes of methadone-maintained former heroin addicts. Neuropsychopharmacology: official publication of the American College of Neuropsychopharmacology..

[15] Nielsen DA (2010). Ethnic diversity of DNA methylation in the OPRM1 promoter region in lymphocytes of heroin addicts. Human genetics..

[16] Ebrahimi, G. *et al*. Elevated levels of DNA methylation at the OPRM1 promoter region in men with opioid use disorder. *The American journal of drug and alcohol abuse* 1–7 (2017). 10.1080/00952990.2016.1275659 28121474

[17] Oertel BG (2012). Genetic-epigenetic interaction modulates mu-opioid receptor regulation. Human molecular genetics..

[18] Zhang H (2012). Hypermethylation of OPRM1 promoter region in European Americans with alcohol dependence. Journal of human genetics..

[19] Knothe C (2016). Pharmacoepigenetics of the role of DNA methylation in mu-opioid receptor expression in different human brain regions. Epigenomics..

[20] Marie-Claire C (2016). Variability of response to methadone: genome-wide DNA methylation analysis in two independent cohorts. Epigenomics..

[21] Gelernter J (2014). Genome-wide association study of cocaine dependence and related traits: FAM53B identified as a risk gene. Molecular psychiatry..

[22] Gelernter J (2015). Genome-wide association study of nicotine dependence in American populations: identification of novel risk loci in both African-Americans and European-Americans. Biological psychiatry..

[23] Gelernter J (2014). Genome-wide association study of alcohol dependence:significant findings in African- and European-Americans including novel risk loci. Molecular psychiatry..

[24] Aryee MJ (2014). Minfi: a flexible and comprehensive Bioconductor package for the analysis of Infinium DNA methylation microarrays. Bioinformatics..

[25] Leek JT, Johnson WE, Parker HS, Jaffe AE, Storey JD (2012). The sva package for removing batch effects and other unwanted variation in high-throughput experiments. Bioinformatics..

[26] Fortin JP (2014). Functional normalization of 450 k methylation array data improves replication in large cancer studies. Genome biology..

[27] Jaffe AE, Irizarry RA (2014). Accounting for cellular heterogeneity is critical in epigenome-wide association studies. Genome biology..

[28] Houseman, E. A. *et al*. DNA methylation arrays as surrogate measures of cell mixture distribution. *BMC bioinformatics* 86 (2012). 10.1186/1471-2105-13-86 PMC3532182 22568884

[29] Barfield RT (2014). Accounting for population stratification in DNA methylation studies. Genet Epidemiol..

[30] Barfield RT, Kilaru V, Smith AK, Conneely KN (2012). CpGassoc: an R function for analysis of DNA methylation microarray data. Bioinformatics..

[31] Joehanes R (2016). Epigenetic Signatures of Cigarette Smoking. Circ Cardiovasc Genet..

[32] Chang, C. C. *et al*. Second-generation PLINK: rising to the challenge of larger and richer datasets. *Gigascience*. 7 (2015). 10.1186/s13742-015-0047-8 PMC4342193 25722852

[33] Cross-Disorder Group of the Psychiatric Genomics C (2013). Identification of risk loci with shared effects on five major psychiatric disorders: a genome-wide analysis. Lancet..

[34] Schizophrenia Psychiatric Genome-Wide Association Study C (2011). Genome-wide association study identifies five new schizophrenia loci. Nature genetics..

[35] Li Z (2017). Genome-wide association analysis identifies 30 new susceptibility loci for schizophrenia. Nature genetics..

[36] Okbay A (2016). Genome-wide association study identifies 74 loci associated with educational attainment. Nature..

[37] Hein MY (2015). A human interactome in three quantitative dimensions organized by stoichiometries and abundances. Cell..

[38] Kozlenkov, A. *et al*. DNA Methylation Profiling of Human Prefrontal Cortex Neurons in Heroin Users Shows Significant Difference between Genomic Contexts of Hyper- and Hypomethylation and a Younger Epigenetic Age. *Genes*. **6** (2017). 10.3390/genes8060152 PMC5485516 28556790

