# Author Correction: Genomewide Study of Epigenetic Biomarkers of Opioid Dependence in European- American Women

**DOI:** 10.1038/s41598-019-55022-z

**Published:** 2019-12-05

**Authors:** Janitza L. Montalvo-Ortiz, Zhongshan Cheng, Henry R. Kranzler, Huiping Zhang, Joel Gelernter

**Affiliations:** 1 Division of Human Genetics, Department of Psychiatry, Yale University School of Medicine, New Haven, CT USA; 2 VA CT Healthcare Center, West Haven, CT USA; 3 University of Pennsylvania Perelman School of Medicine, Department of Psychiatry, Center for Studies of Addiction and Crescenz Veterans Afairs Medical Center, Philadelphia, PA USA; 4 Departments of Psychiatry and Medicine (Biomedical Genetics), Boston University School of Medicine, Boston, MA USA; 5 Departments of Genetics and Neuroscience, Yale University School of Medicine, New Haven, USA

Correction to: *Scientific Reports* 10.1038/s41598-019-41110-7, published online 15 March 2019

This Article contains an error in the Figure legends of Figure 3 and Figure 4. The legends of these Figures were inadvertently switched.

The legend of Figure 3:

“Association between genotype data at rs2611513 and DNA methylation levels at GWS CpG site cg17426237. rs2611513 associated with DNA methylation levels at cg17426237; C allele associated with lower DNA methylation in (**A**) all sample (*n* = 141, *p* = 0.025), and (**B**) OD cases (*n* = 112; *p* = 0.042). No association was obtained in (**C**) opioid-exposed controls (*n* = 29; NS). ^*^Represents *p* < 0.05.”

should read:

“Association between genome-wide significant (GWS) CpG sites and opioid dependence (OD)-related traits. DNA methylation (beta values) of GWS CpG sites associated with OD-related traits are shown: (**A**–**C)** OD symptoms, (**D**–**F)** age of onset (years), (**G**–**I**) duration of opioid use (years), (**J**–**L)** longest duration of chronic opioid use (years). Significant threshold is set at *p* < 0.05.”

The legend of Figure 4:

“Association between genome-wide significant (GWS) CpG sites and opioid dependence (OD)-related traits. DNA methylation (beta values) of GWS CpG sites associated with OD-related traits are shown: (**A**–**C)** OD symptoms, (**D**–**F)** age of onset (years), (**G**–**I**) duration of opioid use (years), (**J**–**L)** longest duration of chronic opioid use (years). Significant threshold is set at *p* < 0.05.”

should read:

“Association between genotype data at rs2611513 and DNA methylation levels at GWS CpG site cg17426237. rs2611513 associated with DNA methylation levels at cg17426237; C allele associated with lower DNA methylation in (**A**) all sample (*n* = 141, *p* = 0.025), and (**B**) OD cases (*n* = 112; *p* = 0.042). No association was obtained in (**C**) opioid-exposed controls (*n* = 29; NS). ^*^Represents *p* < 0.05.”

