# Supplementary material for: Genomewide Study of Epigenetic Biomarkers of Opioid Dependence in European- American Women
Source: Sci Rep. 2019 Mar 15;9:4660. doi: 10.1038/s41598-019-41110-7 (PMC6420601; doi:10.1038/s41598-019-41110-7)
Supplement: Supplementary file 1 — Supplementary Information [file 41598_2019_41110_MOESM1_ESM.docx]

Genomewide Study of Epigenetic Biomarkers of Opioid Dependence in European- American Women

Janitza L. Montalvo-Ortiz^1,2^, Zhongshan Cheng^1,2^, Henry R. Kranzler^3^,

Huiping Zhang^4^, Joel Gelernter^1,2,5^

*Author Affiliations:*

^1^Division of Human Genetics, Department of Psychiatry, Yale University School of Medicine, New Haven, CT; ^2^VA CT Healthcare Center, West Haven, CT; ^3^University of Pennsylvania Perelman School of Medicine, Department of Psychiatry, Center for Studies of Addiction and Crescenz Veterans Affairs Medical Center, Philadelphia, PA; ^4^Departments of Psychiatry and Medicine (Biomedical Genetics), Boston University School of Medicine, Boston, MA; ^5^Departments of Genetics and Neuroscience, Yale University School of Medicine.

*Corresponding Author:*

Joel Gelernter, Yale University School of Medicine, Department of Psychiatry, VA CT 116A2; 950 Campbell Avenue; West Haven, CT 06516; email, [joel.gelernter@yale.edu](mailto:joel.gelernter@yale.edu)

*Short title:* Epigenomics of Opioid Dependence in Women

**Supplementary methods.**

*Power analysis*. G*Power 3.1^1^ was used to compute the power in a Wilcoxon-Mann-Whitney two-group test. Using a moderate effect size (0.5-0.8) based on previous genome-wide DNA methylation studies from our group and others with similar sample sizes^2,3^, we estimated that at genome-wide significance, a sample size of 218 has 60% power in a case-control design (Supplementary Figure 6).

*Brain-blood methylation patterns.* To estimate the brain-blood correlation we used the online version of Blood Brain DNA Methylation Comparison Tool (epigenetics.iopkcl.ac.uk/bloodbrain/) from a previously published study^4^. The correlation in blood with four brain regions (prefrontal cortex, entorhinal cortex, superior temporal gyrus, and cerebellum) was performed for GWS CpG sites.

*Brain-blood gene expression patterns.* To determine the expression patterns of genes from GWS CpG sites across multiple brain regions and whole blood tissue, we used the Genotype-Tissue Expression (GTEx) Project Database portal ([www.gtexportal.org](http://www.gtexportal.org)).

*DNA Methylation Age.* DNA methylation age (DNAm age), a measure of accelerated epigenetic aging, was calculated for each individual based on peripheral blood profiled with the Illumina Infinium EPIC array following the Horvath age estimation algorithm^5^. DNAm age is defined as a multivariate linear model for predicting age based on the methylation levels of 353 CpGs from the array. Epigenetic age acceleration was calculated using publicly available R scripts and a web-based calculator^5^ that uses an elastic net-penalized regression model and provides an estimate (in years) by regressing DNAm age on chronological age, resulting in residuals that can be compared between groups.

**Supplementary Table 1. Summary statistics of OD EWAS analysis (*P*<0.0001).**

| **Illumina ID** | **F statistic** | **FDR** | **P value** |
| --- | --- | --- | --- |
| cg17426237 | 36.70099447 | 0.005316334 | 6.77E-09 |
| cg21381136 | 33.48796732 | 0.021632365 | 2.75E-08 |
| cg18177613 | 31.76346479 | 0.046378533 | 5.90E-08 |
| cg23095642 | 29.05672223 | 0.155669992 | 1.98E-07 |
| cg04983519 | 28.5201434 | 0.19831626 | 2.52E-07 |
| cg24210426 | 28.47736701 | 0.202187529 | 2.57E-07 |
| cg20960678 | 28.1044672 | 0.239347539 | 3.04E-07 |
| cg11395372 | 27.95131516 | 0.256546719 | 3.26E-07 |
| cg05157272 | 27.61388015 | 0.298990092 | 3.80E-07 |
| cg04379122 | 27.5418209 | 0.308938336 | 3.92E-07 |
| cg16255462 | 26.8214773 | 0.428829649 | 5.45E-07 |
| cg10058885 | 26.8113021 | 0.430824652 | 5.47E-07 |
| cg23650853 | 26.66611784 | 0.460335563 | 5.85E-07 |
| cg20788115 | 26.2929123 | 0.545943271 | 6.93E-07 |
| cg23279878 | 26.24457193 | 0.558153165 | 7.09E-07 |
| cg01919034 | 26.13774623 | 0.586125179 | 7.44E-07 |
| cg18407168 | 25.94478993 | 0.640298834 | 8.13E-07 |
| cg14911807 | 25.79712735 | 0.685159063 | 8.70E-07 |
| cg00395929 | 24.90497892 | 1 | 1.31E-06 |
| cg21787216 | 24.86210653 | 1 | 1.34E-06 |
| cg09108314 | 24.83026071 | 1 | 1.36E-06 |
| cg26799806 | 24.71057607 | 1 | 1.43E-06 |
| cg19332831 | 24.6877498 | 1 | 1.45E-06 |
| cg09556412 | 24.62280929 | 1 | 1.49E-06 |
| cg06055730 | 24.60510432 | 1 | 1.51E-06 |
| cg05675926 | 24.54762436 | 1 | 1.55E-06 |
| cg27022602 | 24.46505311 | 1 | 1.61E-06 |
| cg16516670 | 24.44518033 | 1 | 1.62E-06 |
| cg05013825 | 24.1404443 | 1 | 1.87E-06 |
| cg14832223 | 24.06346141 | 1 | 1.93E-06 |
| cg08298766 | 24.03189036 | 1 | 1.96E-06 |
| cg16295231 | 23.98098201 | 1 | 2.01E-06 |
| cg01341130 | 23.92924503 | 1 | 2.06E-06 |
| cg10476980 | 23.91658406 | 1 | 2.07E-06 |
| cg25837984 | 23.89898042 | 1 | 2.09E-06 |
| cg20114879 | 23.84305988 | 1 | 2.14E-06 |
| cg24808111 | 23.76001527 | 1 | 2.23E-06 |
| cg18876623 | 23.75373282 | 1 | 2.23E-06 |
| cg20559736 | 23.53456488 | 1 | 2.47E-06 |
| cg04255814 | 23.46385165 | 1 | 2.55E-06 |
| cg21453477 | 23.43891432 | 1 | 2.58E-06 |
| cg18866197 | 23.33102991 | 1 | 2.72E-06 |
| cg12456794 | 23.31092512 | 1 | 2.74E-06 |
| cg24706124 | 23.17909552 | 1 | 2.92E-06 |
| cg07801516 | 23.13617714 | 1 | 2.97E-06 |
| cg19797341 | 23.10574939 | 1 | 3.02E-06 |
| cg08739385 | 22.96104169 | 1 | 3.23E-06 |
| cg27245056 | 22.88335733 | 1 | 3.35E-06 |
| cg23675000 | 22.83482584 | 1 | 3.42E-06 |
| cg01169979 | 22.82794783 | 1 | 3.43E-06 |
| cg09832197 | 22.79739616 | 1 | 3.48E-06 |
| cg11022537 | 22.7575769 | 1 | 3.55E-06 |
| cg13272676 | 22.74490735 | 1 | 3.57E-06 |
| cg25260828 | 22.74160449 | 1 | 3.58E-06 |
| cg01897092 | 22.71648003 | 1 | 3.62E-06 |
| cg25477905 | 22.59784226 | 1 | 3.82E-06 |
| cg16323212 | 22.57084394 | 1 | 3.87E-06 |
| cg11024200 | 22.55018702 | 1 | 3.91E-06 |
| cg03057182 | 22.31969901 | 1 | 4.36E-06 |
| cg24597774 | 22.30996311 | 1 | 4.38E-06 |
| cg21233275 | 22.23107112 | 1 | 4.54E-06 |
| cg03256616 | 22.06421976 | 1 | 4.91E-06 |
| cg20797552 | 22.06169921 | 1 | 4.91E-06 |
| cg00076936 | 21.93229558 | 1 | 5.22E-06 |
| cg02708544 | 21.868228 | 1 | 5.38E-06 |
| cg11249066 | 21.86184161 | 1 | 5.40E-06 |
| cg10279055 | 21.85054808 | 1 | 5.43E-06 |
| cg09580755 | 21.78497272 | 1 | 5.60E-06 |
| cg09130192 | 21.75161714 | 1 | 5.68E-06 |
| cg25621735 | 21.70950972 | 1 | 5.80E-06 |
| cg08071689 | 21.63949644 | 1 | 5.99E-06 |
| cg05402288 | 21.62798509 | 1 | 6.02E-06 |
| cg17840051 | 21.60094047 | 1 | 6.10E-06 |
| cg14867639 | 21.56028643 | 1 | 6.22E-06 |
| cg27021666 | 21.5193089 | 1 | 6.34E-06 |
| cg13286692 | 21.5157297 | 1 | 6.35E-06 |
| cg06600200 | 21.49039007 | 1 | 6.43E-06 |
| cg03032574 | 21.47496699 | 1 | 6.47E-06 |
| cg19656847 | 21.45242404 | 1 | 6.54E-06 |
| cg09736124 | 21.45180313 | 1 | 6.55E-06 |
| cg24646457 | 21.41791014 | 1 | 6.65E-06 |
| cg27227597 | 21.41230072 | 1 | 6.67E-06 |
| cg18424706 | 21.4034081 | 1 | 6.70E-06 |
| cg22815999 | 21.39795262 | 1 | 6.71E-06 |
| cg05869737 | 21.39699852 | 1 | 6.72E-06 |
| cg26506792 | 21.37492068 | 1 | 6.79E-06 |
| cg08035151 | 21.32694146 | 1 | 6.94E-06 |
| cg10130460 | 21.30741784 | 1 | 7.01E-06 |
| cg17309592 | 21.2940134 | 1 | 7.05E-06 |
| cg03760759 | 21.22796133 | 1 | 7.27E-06 |
| cg24986225 | 21.20790503 | 1 | 7.34E-06 |
| cg06624244 | 21.18362543 | 1 | 7.43E-06 |
| cg01336552 | 21.0962617 | 1 | 7.74E-06 |
| cg26498546 | 21.08509865 | 1 | 7.78E-06 |
| cg26088753 | 21.06574967 | 1 | 7.85E-06 |
| cg16698828 | 21.04001421 | 1 | 7.95E-06 |
| cg08464860 | 21.01805441 | 1 | 8.03E-06 |
| cg10243170 | 20.98166192 | 1 | 8.17E-06 |
| cg08164950 | 20.97797615 | 1 | 8.18E-06 |
| cg26924044 | 20.96287828 | 1 | 8.24E-06 |
| cg08101859 | 20.95494807 | 1 | 8.27E-06 |
| cg21813478 | 20.91366942 | 1 | 8.44E-06 |
| cg25340121 | 20.91084477 | 1 | 8.45E-06 |
| cg19479828 | 20.88688528 | 1 | 8.54E-06 |
| cg14590262 | 20.88333099 | 1 | 8.56E-06 |
| cg10819560 | 20.85347988 | 1 | 8.68E-06 |
| cg07427336 | 20.77023455 | 1 | 9.03E-06 |
| cg02449575 | 20.72736777 | 1 | 9.21E-06 |
| cg23580358 | 20.6963476 | 1 | 9.35E-06 |
| cg14548038 | 20.69242717 | 1 | 9.36E-06 |
| cg07060960 | 20.67339341 | 1 | 9.45E-06 |
| cg00954996 | 20.56895692 | 1 | 9.93E-06 |
| cg09472784 | 20.54438486 | 1 | 1.00E-05 |
| cg05118364 | 20.50905967 | 1 | 1.02E-05 |
| cg17854641 | 20.49801838 | 1 | 1.03E-05 |
| cg04169102 | 20.49247069 | 1 | 1.03E-05 |
| cg05919975 | 20.4726826 | 1 | 1.04E-05 |
| cg09766849 | 20.46755563 | 1 | 1.04E-05 |
| cg00941856 | 20.45956774 | 1 | 1.05E-05 |
| cg00225587 | 20.4593105 | 1 | 1.05E-05 |
| cg19120943 | 20.44388244 | 1 | 1.05E-05 |
| cg23666072 | 20.42818809 | 1 | 1.06E-05 |
| cg21274724 | 20.41577685 | 1 | 1.07E-05 |
| cg05965444 | 20.40010882 | 1 | 1.08E-05 |
| cg02739641 | 20.35429557 | 1 | 1.10E-05 |
| cg20971342 | 20.35062077 | 1 | 1.10E-05 |
| cg01866220 | 20.34550541 | 1 | 1.10E-05 |
| cg06775669 | 20.33237549 | 1 | 1.11E-05 |
| cg04253037 | 20.32974463 | 1 | 1.11E-05 |
| cg02996092 | 20.31284199 | 1 | 1.12E-05 |
| cg24646027 | 20.29319106 | 1 | 1.13E-05 |
| cg00321115 | 20.28235256 | 1 | 1.14E-05 |
| cg22496450 | 20.27123114 | 1 | 1.14E-05 |
| cg13916146 | 20.25823627 | 1 | 1.15E-05 |
| cg22414994 | 20.19840974 | 1 | 1.18E-05 |
| cg21153648 | 20.18768455 | 1 | 1.19E-05 |
| cg07132926 | 20.18764347 | 1 | 1.19E-05 |
| cg22619628 | 20.16446261 | 1 | 1.20E-05 |
| cg23554506 | 20.13316153 | 1 | 1.22E-05 |
| cg00399861 | 20.12320189 | 1 | 1.23E-05 |
| cg25515854 | 20.10884155 | 1 | 1.23E-05 |
| cg16762000 | 20.0947791 | 1 | 1.24E-05 |
| cg01195526 | 20.0600449 | 1 | 1.26E-05 |
| cg14630164 | 20.05627799 | 1 | 1.27E-05 |
| cg11913696 | 20.04615384 | 1 | 1.27E-05 |
| cg05587970 | 19.98912843 | 1 | 1.31E-05 |
| cg05271080 | 19.9886265 | 1 | 1.31E-05 |
| cg22717779 | 19.96762656 | 1 | 1.32E-05 |
| cg27375351 | 19.94383927 | 1 | 1.34E-05 |
| cg14028022 | 19.92890591 | 1 | 1.34E-05 |
| cg24290299 | 19.9287097 | 1 | 1.35E-05 |
| cg14331998 | 19.91868166 | 1 | 1.35E-05 |
| cg20972078 | 19.91185591 | 1 | 1.36E-05 |
| cg18467339 | 19.90496841 | 1 | 1.36E-05 |
| cg03551378 | 19.88159363 | 1 | 1.38E-05 |
| cg16396593 | 19.86049705 | 1 | 1.39E-05 |
| cg21059945 | 19.84129163 | 1 | 1.40E-05 |
| cg06991713 | 19.82695056 | 1 | 1.41E-05 |
| cg05479988 | 19.82471407 | 1 | 1.41E-05 |
| cg13445938 | 19.82308723 | 1 | 1.41E-05 |
| cg24587796 | 19.82252667 | 1 | 1.41E-05 |
| cg25758587 | 19.80622468 | 1 | 1.43E-05 |
| cg15619333 | 19.79730183 | 1 | 1.43E-05 |
| cg22794712 | 19.78478982 | 1 | 1.44E-05 |
| cg12340314 | 19.78286798 | 1 | 1.44E-05 |
| cg04120002 | 19.7753757 | 1 | 1.45E-05 |
| cg00512337 | 19.72543356 | 1 | 1.48E-05 |
| cg27461942 | 19.70405679 | 1 | 1.50E-05 |
| cg26095478 | 19.69018227 | 1 | 1.51E-05 |
| cg04446983 | 19.67271813 | 1 | 1.52E-05 |
| cg10815395 | 19.65644777 | 1 | 1.53E-05 |
| cg07834934 | 19.60286374 | 1 | 1.57E-05 |
| cg09246924 | 19.6000127 | 1 | 1.57E-05 |
| cg20146387 | 19.56315322 | 1 | 1.60E-05 |
| cg03537567 | 19.51098213 | 1 | 1.64E-05 |
| cg10194352 | 19.48717494 | 1 | 1.66E-05 |
| cg07067773 | 19.46831308 | 1 | 1.67E-05 |
| cg02775882 | 19.45693558 | 1 | 1.68E-05 |
| cg16685832 | 19.44984119 | 1 | 1.69E-05 |
| cg11966886 | 19.42855878 | 1 | 1.71E-05 |
| cg02019955 | 19.36999466 | 1 | 1.75E-05 |
| cg04664860 | 19.36585467 | 1 | 1.76E-05 |
| cg01409552 | 19.36073701 | 1 | 1.76E-05 |
| cg19190632 | 19.35322603 | 1 | 1.77E-05 |
| cg15538009 | 19.32683401 | 1 | 1.79E-05 |
| cg21942282 | 19.32534276 | 1 | 1.79E-05 |
| cg26867282 | 19.32377362 | 1 | 1.79E-05 |
| cg20646016 | 19.3089795 | 1 | 1.81E-05 |
| cg24078554 | 19.29429582 | 1 | 1.82E-05 |
| cg24400553 | 19.19766697 | 1 | 1.91E-05 |
| cg16476639 | 19.17693749 | 1 | 1.92E-05 |
| cg10717504 | 19.17252036 | 1 | 1.93E-05 |
| cg18873878 | 19.1619435 | 1 | 1.94E-05 |
| cg14701887 | 19.15540227 | 1 | 1.94E-05 |
| cg05445097 | 19.14201646 | 1 | 1.96E-05 |
| cg20245561 | 19.09656351 | 1 | 2.00E-05 |
| cg23571990 | 19.05673914 | 1 | 2.04E-05 |
| cg03076246 | 19.04685675 | 1 | 2.05E-05 |
| cg26789319 | 19.04058916 | 1 | 2.05E-05 |
| cg18311854 | 19.02840714 | 1 | 2.07E-05 |
| cg06157388 | 19.01948948 | 1 | 2.07E-05 |
| cg26609894 | 18.99623764 | 1 | 2.10E-05 |
| cg24354101 | 18.98720315 | 1 | 2.11E-05 |
| cg27415214 | 18.98630535 | 1 | 2.11E-05 |
| cg16684608 | 18.98321416 | 1 | 2.11E-05 |
| cg08407553 | 18.96780609 | 1 | 2.13E-05 |
| cg17104615 | 18.90369694 | 1 | 2.19E-05 |
| cg07007780 | 18.8859316 | 1 | 2.21E-05 |
| cg09244983 | 18.87485852 | 1 | 2.22E-05 |
| cg26673151 | 18.86385601 | 1 | 2.24E-05 |
| cg22580103 | 18.86120579 | 1 | 2.24E-05 |
| cg16796215 | 18.84589837 | 1 | 2.25E-05 |
| cg12216772 | 18.83011915 | 1 | 2.27E-05 |
| cg04013419 | 18.82266661 | 1 | 2.28E-05 |
| cg15834198 | 18.79956828 | 1 | 2.30E-05 |
| cg02373061 | 18.76395386 | 1 | 2.34E-05 |
| cg06869501 | 18.73832081 | 1 | 2.37E-05 |
| cg12985204 | 18.73108912 | 1 | 2.38E-05 |
| cg26325497 | 18.72260195 | 1 | 2.39E-05 |
| cg25221988 | 18.70368725 | 1 | 2.41E-05 |
| cg05479174 | 18.66656908 | 1 | 2.46E-05 |
| cg00614413 | 18.64032231 | 1 | 2.49E-05 |
| cg19270118 | 18.61780855 | 1 | 2.51E-05 |
| cg00920668 | 18.59391613 | 1 | 2.54E-05 |
| cg00764840 | 18.58004488 | 1 | 2.56E-05 |
| cg25437757 | 18.57446717 | 1 | 2.57E-05 |
| cg05982817 | 18.57212534 | 1 | 2.57E-05 |
| cg15866047 | 18.56679996 | 1 | 2.58E-05 |
| cg27361617 | 18.55488875 | 1 | 2.59E-05 |
| cg09371661 | 18.54047234 | 1 | 2.61E-05 |
| cg02780400 | 18.53111322 | 1 | 2.62E-05 |
| cg11180306 | 18.50287749 | 1 | 2.66E-05 |
| cg19987349 | 18.45158683 | 1 | 2.72E-05 |
| cg02999390 | 18.44522443 | 1 | 2.73E-05 |
| cg03689540 | 18.43576075 | 1 | 2.74E-05 |
| cg19086488 | 18.42320455 | 1 | 2.76E-05 |
| cg04797274 | 18.4177145 | 1 | 2.77E-05 |
| cg16396580 | 18.40846994 | 1 | 2.78E-05 |
| cg22253168 | 18.40418627 | 1 | 2.79E-05 |
| cg08529864 | 18.3846703 | 1 | 2.81E-05 |
| cg21545248 | 18.38196119 | 1 | 2.82E-05 |
| cg01217180 | 18.3760702 | 1 | 2.82E-05 |
| cg17514665 | 18.36696764 | 1 | 2.84E-05 |
| cg01335738 | 18.36594939 | 1 | 2.84E-05 |
| cg16526479 | 18.33337745 | 1 | 2.88E-05 |
| cg14038259 | 18.31772198 | 1 | 2.90E-05 |
| cg14168958 | 18.29505468 | 1 | 2.94E-05 |
| cg16797860 | 18.28503769 | 1 | 2.95E-05 |
| cg13048147 | 18.2477466 | 1 | 3.00E-05 |
| cg09510269 | 18.24663393 | 1 | 3.01E-05 |
| cg10472320 | 18.21300623 | 1 | 3.05E-05 |
| cg02474109 | 18.19615318 | 1 | 3.08E-05 |
| cg05671511 | 18.19271405 | 1 | 3.08E-05 |
| cg22232320 | 18.19104072 | 1 | 3.09E-05 |
| cg08453814 | 18.15181384 | 1 | 3.15E-05 |
| cg10955297 | 18.14013527 | 1 | 3.16E-05 |
| cg13223329 | 18.13036082 | 1 | 3.18E-05 |
| cg11157324 | 18.09823245 | 1 | 3.23E-05 |
| cg10761141 | 18.08792036 | 1 | 3.24E-05 |
| cg21839284 | 18.0622448 | 1 | 3.28E-05 |
| cg20149531 | 18.04997897 | 1 | 3.30E-05 |
| cg15700971 | 18.04280552 | 1 | 3.31E-05 |
| cg24611996 | 18.03894033 | 1 | 3.32E-05 |
| cg11111835 | 17.99494902 | 1 | 3.39E-05 |
| cg20347042 | 17.99325663 | 1 | 3.39E-05 |
| cg00044440 | 17.95408547 | 1 | 3.46E-05 |
| cg24172570 | 17.94421048 | 1 | 3.48E-05 |
| cg11704463 | 17.93977438 | 1 | 3.48E-05 |
| cg21742728 | 17.93405175 | 1 | 3.49E-05 |
| cg01685977 | 17.93028908 | 1 | 3.50E-05 |
| cg06916404 | 17.91229902 | 1 | 3.53E-05 |
| cg10832417 | 17.90608926 | 1 | 3.54E-05 |
| cg22546437 | 17.89603065 | 1 | 3.56E-05 |
| cg02240671 | 17.88741012 | 1 | 3.57E-05 |
| cg23354802 | 17.88640709 | 1 | 3.57E-05 |
| cg10337500 | 17.86843569 | 1 | 3.61E-05 |
| cg10625354 | 17.85526414 | 1 | 3.63E-05 |
| cg27275723 | 17.84473238 | 1 | 3.65E-05 |
| cg22840251 | 17.79416362 | 1 | 3.74E-05 |
| cg14132357 | 17.78021574 | 1 | 3.76E-05 |
| cg14939641 | 17.77104856 | 1 | 3.78E-05 |
| cg04505205 | 17.72862974 | 1 | 3.86E-05 |
| cg06215939 | 17.69753103 | 1 | 3.92E-05 |
| cg02963265 | 17.67409752 | 1 | 3.96E-05 |
| cg02528144 | 17.64593993 | 1 | 4.01E-05 |
| cg24688939 | 17.64490562 | 1 | 4.02E-05 |
| cg22385669 | 17.61994032 | 1 | 4.06E-05 |
| cg22606564 | 17.61280377 | 1 | 4.08E-05 |
| cg03047400 | 17.60112834 | 1 | 4.10E-05 |
| cg04865926 | 17.59337994 | 1 | 4.12E-05 |
| cg24941977 | 17.58496876 | 1 | 4.13E-05 |
| cg01409850 | 17.58379393 | 1 | 4.14E-05 |
| cg10701168 | 17.57297144 | 1 | 4.16E-05 |
| cg01009697 | 17.56599023 | 1 | 4.17E-05 |
| cg15326454 | 17.56089793 | 1 | 4.18E-05 |
| cg08546856 | 17.55959571 | 1 | 4.18E-05 |
| cg26945748 | 17.54969787 | 1 | 4.20E-05 |
| cg17106686 | 17.5397889 | 1 | 4.22E-05 |
| cg17846723 | 17.52862322 | 1 | 4.25E-05 |
| cg15403961 | 17.5271337 | 1 | 4.25E-05 |
| cg04117460 | 17.51901412 | 1 | 4.27E-05 |
| cg24108888 | 17.5060441 | 1 | 4.29E-05 |
| cg11116176 | 17.49434096 | 1 | 4.32E-05 |
| cg23101469 | 17.49347186 | 1 | 4.32E-05 |
| cg17383884 | 17.48814616 | 1 | 4.33E-05 |
| cg02236489 | 17.48260239 | 1 | 4.34E-05 |
| cg20185525 | 17.46297266 | 1 | 4.38E-05 |
| cg10219156 | 17.4514495 | 1 | 4.41E-05 |
| cg09535795 | 17.443839 | 1 | 4.43E-05 |
| cg00166834 | 17.43911281 | 1 | 4.44E-05 |
| cg07590922 | 17.43492172 | 1 | 4.44E-05 |
| cg27485605 | 17.43303496 | 1 | 4.45E-05 |
| cg05381183 | 17.42501761 | 1 | 4.47E-05 |
| cg08958656 | 17.39752771 | 1 | 4.53E-05 |
| cg16559448 | 17.38265599 | 1 | 4.56E-05 |
| cg24918985 | 17.3231034 | 1 | 4.69E-05 |
| cg25395945 | 17.3168198 | 1 | 4.71E-05 |
| cg11748170 | 17.29978057 | 1 | 4.74E-05 |
| cg00415763 | 17.29463433 | 1 | 4.76E-05 |
| cg24198209 | 17.28032722 | 1 | 4.79E-05 |
| cg23237765 | 17.26639869 | 1 | 4.82E-05 |
| cg18647237 | 17.26546827 | 1 | 4.82E-05 |
| cg19689198 | 17.24820167 | 1 | 4.86E-05 |
| cg06029627 | 17.22890744 | 1 | 4.91E-05 |
| cg03230491 | 17.2226172 | 1 | 4.93E-05 |
| cg09246385 | 17.2050257 | 1 | 4.97E-05 |
| cg04197935 | 17.20152085 | 1 | 4.98E-05 |
| cg10365657 | 17.18284031 | 1 | 5.02E-05 |
| cg19344049 | 17.1819133 | 1 | 5.02E-05 |
| cg03472610 | 17.17875362 | 1 | 5.03E-05 |
| cg25950438 | 17.17116 | 1 | 5.05E-05 |
| cg07397958 | 17.16125177 | 1 | 5.07E-05 |
| cg11545863 | 17.15910064 | 1 | 5.08E-05 |
| cg02294570 | 17.15787289 | 1 | 5.08E-05 |
| cg09613050 | 17.15228654 | 1 | 5.10E-05 |
| cg15956369 | 17.14365383 | 1 | 5.12E-05 |
| cg20036711 | 17.11895691 | 1 | 5.18E-05 |
| cg27228191 | 17.11673071 | 1 | 5.18E-05 |
| cg02181187 | 17.10617543 | 1 | 5.21E-05 |
| cg11415852 | 17.05652385 | 1 | 5.34E-05 |
| cg10452974 | 17.05151551 | 1 | 5.35E-05 |
| cg19730523 | 17.04230868 | 1 | 5.38E-05 |
| cg03937717 | 17.0399801 | 1 | 5.38E-05 |
| cg11877440 | 17.03694732 | 1 | 5.39E-05 |
| ch.13.63716R | 17.03610196 | 1 | 5.39E-05 |
| cg12953445 | 17.03488146 | 1 | 5.39E-05 |
| cg07771804 | 17.02531857 | 1 | 5.42E-05 |
| cg07057342 | 17.01950533 | 1 | 5.43E-05 |
| cg18344056 | 17.00028509 | 1 | 5.49E-05 |
| cg04666976 | 16.99800612 | 1 | 5.49E-05 |
| cg15388570 | 16.99192187 | 1 | 5.51E-05 |
| cg09915978 | 16.97973865 | 1 | 5.54E-05 |
| cg17089785 | 16.96676845 | 1 | 5.58E-05 |
| cg04468334 | 16.95426143 | 1 | 5.61E-05 |
| cg19870125 | 16.94901771 | 1 | 5.62E-05 |
| cg26117521 | 16.94693079 | 1 | 5.63E-05 |
| cg27574374 | 16.94327976 | 1 | 5.64E-05 |
| cg03100611 | 16.93967279 | 1 | 5.65E-05 |
| cg10259643 | 16.93059173 | 1 | 5.67E-05 |
| cg12439834 | 16.90438904 | 1 | 5.75E-05 |
| cg14028272 | 16.88428046 | 1 | 5.80E-05 |
| cg24342565 | 16.88421564 | 1 | 5.80E-05 |
| cg01709096 | 16.87694304 | 1 | 5.82E-05 |
| cg22772296 | 16.85549482 | 1 | 5.89E-05 |
| cg13799544 | 16.84247236 | 1 | 5.92E-05 |
| cg17829914 | 16.84050186 | 1 | 5.93E-05 |
| cg08314899 | 16.83616483 | 1 | 5.94E-05 |
| cg11450744 | 16.82886793 | 1 | 5.96E-05 |
| cg21113846 | 16.82077146 | 1 | 5.99E-05 |
| cg18203393 | 16.81972219 | 1 | 5.99E-05 |
| cg26508775 | 16.81510721 | 1 | 6.00E-05 |
| cg07898303 | 16.80540173 | 1 | 6.03E-05 |
| cg02144030 | 16.77353746 | 1 | 6.12E-05 |
| cg05804856 | 16.7708761 | 1 | 6.13E-05 |
| cg26332016 | 16.7619613 | 1 | 6.16E-05 |
| cg00300233 | 16.75997534 | 1 | 6.16E-05 |
| cg10006535 | 16.7515301 | 1 | 6.19E-05 |
| cg06624731 | 16.73166056 | 1 | 6.25E-05 |
| cg14762495 | 16.73084603 | 1 | 6.25E-05 |
| cg20739309 | 16.72491594 | 1 | 6.27E-05 |
| cg17310120 | 16.72381204 | 1 | 6.27E-05 |
| cg15924127 | 16.71967363 | 1 | 6.29E-05 |
| cg21570818 | 16.71479607 | 1 | 6.30E-05 |
| cg03058862 | 16.70979247 | 1 | 6.32E-05 |
| cg09463656 | 16.70380195 | 1 | 6.34E-05 |
| cg16234335 | 16.69557855 | 1 | 6.36E-05 |
| cg22118655 | 16.67806185 | 1 | 6.42E-05 |
| cg16324015 | 16.6686055 | 1 | 6.44E-05 |
| cg16589580 | 16.66746381 | 1 | 6.45E-05 |
| cg12056000 | 16.66571756 | 1 | 6.45E-05 |
| cg08599824 | 16.65830627 | 1 | 6.48E-05 |
| cg02944953 | 16.64489782 | 1 | 6.52E-05 |
| cg22751046 | 16.63337438 | 1 | 6.56E-05 |
| cg20145601 | 16.63002787 | 1 | 6.57E-05 |
| cg19853434 | 16.61817705 | 1 | 6.60E-05 |
| cg02402209 | 16.60920758 | 1 | 6.63E-05 |
| cg26036466 | 16.60498205 | 1 | 6.65E-05 |
| cg10976426 | 16.57779087 | 1 | 6.74E-05 |
| cg02986878 | 16.56446587 | 1 | 6.78E-05 |
| cg05289466 | 16.56113546 | 1 | 6.79E-05 |
| cg07276140 | 16.5558023 | 1 | 6.81E-05 |
| cg13390224 | 16.54232983 | 1 | 6.85E-05 |
| cg23911406 | 16.53947075 | 1 | 6.86E-05 |
| cg09050761 | 16.53665581 | 1 | 6.87E-05 |
| cg23319790 | 16.51654711 | 1 | 6.94E-05 |
| cg15118519 | 16.51602972 | 1 | 6.94E-05 |
| cg16059293 | 16.48153332 | 1 | 7.06E-05 |
| cg21740507 | 16.45672967 | 1 | 7.14E-05 |
| cg25195309 | 16.45339055 | 1 | 7.16E-05 |
| cg07612877 | 16.40057818 | 1 | 7.34E-05 |
| cg12608603 | 16.39519173 | 1 | 7.36E-05 |
| cg19688321 | 16.39417177 | 1 | 7.37E-05 |
| cg00905858 | 16.38654304 | 1 | 7.39E-05 |
| cg14339971 | 16.3797815 | 1 | 7.42E-05 |
| cg07592944 | 16.37302964 | 1 | 7.44E-05 |
| cg14336526 | 16.34464116 | 1 | 7.55E-05 |
| cg15824124 | 16.3403405 | 1 | 7.56E-05 |
| cg22802174 | 16.33785808 | 1 | 7.57E-05 |
| cg17709512 | 16.33063457 | 1 | 7.60E-05 |
| cg02403931 | 16.32341494 | 1 | 7.62E-05 |
| cg09107055 | 16.31650915 | 1 | 7.65E-05 |
| cg03228602 | 16.31202375 | 1 | 7.67E-05 |
| cg06452176 | 16.31073304 | 1 | 7.67E-05 |
| cg19898351 | 16.29894823 | 1 | 7.72E-05 |
| cg15032849 | 16.29787341 | 1 | 7.72E-05 |
| cg24750627 | 16.29725257 | 1 | 7.72E-05 |
| cg24353651 | 16.29403343 | 1 | 7.73E-05 |
| cg04056904 | 16.26766137 | 1 | 7.83E-05 |
| cg20925075 | 16.26355912 | 1 | 7.85E-05 |
| cg15339796 | 16.25991042 | 1 | 7.86E-05 |
| cg04556393 | 16.25780695 | 1 | 7.87E-05 |
| cg22026677 | 16.25430489 | 1 | 7.89E-05 |
| cg15676500 | 16.25237523 | 1 | 7.89E-05 |
| cg01899581 | 16.21518494 | 1 | 8.04E-05 |
| cg02150363 | 16.19762486 | 1 | 8.11E-05 |
| cg08900677 | 16.19658821 | 1 | 8.11E-05 |
| cg23111248 | 16.19649145 | 1 | 8.11E-05 |
| cg02037976 | 16.18870447 | 1 | 8.14E-05 |
| cg05476998 | 16.17665825 | 1 | 8.19E-05 |
| cg04604638 | 16.17596159 | 1 | 8.19E-05 |
| cg22754654 | 16.17583811 | 1 | 8.19E-05 |
| cg17755269 | 16.17407457 | 1 | 8.20E-05 |
| cg10726312 | 16.17162087 | 1 | 8.21E-05 |
| cg19674350 | 16.1699577 | 1 | 8.22E-05 |
| cg27304437 | 16.16434069 | 1 | 8.24E-05 |
| cg03665229 | 16.16075009 | 1 | 8.25E-05 |
| cg00023828 | 16.14961783 | 1 | 8.30E-05 |
| cg17155610 | 16.14855534 | 1 | 8.30E-05 |
| cg13200434 | 16.14307047 | 1 | 8.33E-05 |
| cg16955407 | 16.14256195 | 1 | 8.33E-05 |
| cg11680176 | 16.14205047 | 1 | 8.33E-05 |
| cg17104905 | 16.13346165 | 1 | 8.36E-05 |
| cg07443932 | 16.12693422 | 1 | 8.39E-05 |
| cg15281724 | 16.12323842 | 1 | 8.41E-05 |
| cg15157403 | 16.12033144 | 1 | 8.42E-05 |
| cg00494556 | 16.11737625 | 1 | 8.43E-05 |
| cg14159373 | 16.11565749 | 1 | 8.44E-05 |
| cg16301728 | 16.11469252 | 1 | 8.44E-05 |
| cg08823985 | 16.11435531 | 1 | 8.44E-05 |
| cg25947600 | 16.10997042 | 1 | 8.46E-05 |
| cg12009809 | 16.10357684 | 1 | 8.49E-05 |
| cg01612366 | 16.10196898 | 1 | 8.49E-05 |
| cg04433354 | 16.10127372 | 1 | 8.50E-05 |
| cg08244785 | 16.09545754 | 1 | 8.52E-05 |
| cg26310969 | 16.09342465 | 1 | 8.53E-05 |
| cg10364459 | 16.09219492 | 1 | 8.53E-05 |
| cg23620076 | 16.07812036 | 1 | 8.59E-05 |
| cg02391555 | 16.07570087 | 1 | 8.60E-05 |
| cg22053861 | 16.05411117 | 1 | 8.69E-05 |
| cg16097349 | 16.05403158 | 1 | 8.70E-05 |
| cg18264913 | 16.05268579 | 1 | 8.70E-05 |
| cg08214142 | 16.04558021 | 1 | 8.73E-05 |
| cg01510332 | 16.04233986 | 1 | 8.74E-05 |
| cg14550752 | 16.03928003 | 1 | 8.76E-05 |
| cg03155271 | 16.03676443 | 1 | 8.77E-05 |
| cg02120071 | 16.00569796 | 1 | 8.90E-05 |
| cg05685023 | 16.00528672 | 1 | 8.90E-05 |
| cg02858510 | 16.00001769 | 1 | 8.93E-05 |
| cg03791799 | 15.99891542 | 1 | 8.93E-05 |
| cg07194700 | 15.98324629 | 1 | 9.00E-05 |
| cg25175684 | 15.97470135 | 1 | 9.04E-05 |
| cg14992760 | 15.97291469 | 1 | 9.05E-05 |
| cg27543728 | 15.97285392 | 1 | 9.05E-05 |
| cg01861799 | 15.95366996 | 1 | 9.13E-05 |
| cg09740319 | 15.95336678 | 1 | 9.13E-05 |
| cg24981504 | 15.93891271 | 1 | 9.20E-05 |
| cg07233389 | 15.90585295 | 1 | 9.35E-05 |
| cg07312150 | 15.90175652 | 1 | 9.37E-05 |
| cg05294497 | 15.89845407 | 1 | 9.38E-05 |
| cg22132013 | 15.89578725 | 1 | 9.39E-05 |
| cg25724460 | 15.89026797 | 1 | 9.42E-05 |
| cg20569145 | 15.87754349 | 1 | 9.48E-05 |
| cg10530572 | 15.87480892 | 1 | 9.49E-05 |
| cg22388903 | 15.87429853 | 1 | 9.49E-05 |
| cg18056754 | 15.87416833 | 1 | 9.49E-05 |
| cg11960723 | 15.87302676 | 1 | 9.50E-05 |
| cg19783954 | 15.86379732 | 1 | 9.54E-05 |
| cg21731779 | 15.85540874 | 1 | 9.58E-05 |
| cg24152297 | 15.85361972 | 1 | 9.59E-05 |
| cg09076012 | 15.85316041 | 1 | 9.59E-05 |
| cg27408262 | 15.85312385 | 1 | 9.59E-05 |
| cg05201084 | 15.83566948 | 1 | 9.67E-05 |
| cg07054184 | 15.82277911 | 1 | 9.74E-05 |
| cg13460465 | 15.81749958 | 1 | 9.76E-05 |
| cg24986058 | 15.81065207 | 1 | 9.79E-05 |
| cg14037652 | 15.80485973 | 1 | 9.82E-05 |
| cg23517677 | 15.79312022 | 1 | 9.88E-05 |
| cg11752061 | 15.79099104 | 1 | 9.89E-05 |
| cg24346110 | 15.78795754 | 1 | 9.90E-05 |
| cg13776199 | 15.78703387 | 1 | 9.91E-05 |
| cg01987196 | 15.78643101 | 1 | 9.91E-05 |
| cg07127103 | 15.78477104 | 1 | 9.92E-05 |
| cg23701012 | 15.78242299 | 1 | 9.93E-05 |
| cg06567042 | 15.77602518 | 1 | 9.96E-05 |
| cg17294198 | 15.77516833 | 1 | 9.97E-05 |
| cg27570517 | 15.77098076 | 1 | 9.99E-05 |

**Supplementary Figure 1. Density plots.**

Distribution of beta values before and after normalization.

**Supplementary Figure 2. Quantile-quantile plot.**

Quantile-quantile plot illustrates the *p*-values of the association between DNA methylation and OD. There is no evidence for inflation (λ=1.00).

**Supplementary Figure 3. Brain-blood methylation patterns of GWS CpG sites.**

Publicly available data from Hannon *et al.* was used to assess cg17426237 DNA methylation patterns across multiple tissues: blood, prefrontal cortex (PFC), entorhinal cortex (EC), superior temporal gyrus (STG), and cerebellum (CER).

**Supplementary Figure 4. Brain-blood gene expression patterns of genes from GWS CpG sites.**

Genotype-Tissue Extension (GTEx) data (v.7) was used to assess the gene expression patterns of *PARG* (**A**) and *RERE* (**B**) from cg17426237 and cg21381136, respectively, across multiple brain regions and whole blood tissue.

**Supplementary Figure 5. Relationship between genome-wide significant (GWS) CpG sites and other clinical variables.**

Violin plots show the relationship between DNA methylation levels (beta values) of GWS CpG sites and clinical variables such as **A, D, G)** alcohol dependence, **B, E, H)** cocaine dependence, and **C, F, I)** current smoking.

**Supplementary Figure 6. Epigenetic age acceleration in peripheral blood of opioid-dependent subjects and opioid-exposed controls.**

Correlation of DNAm age with chronological age of all subjects. (**B**) Epigenetic age acceleration, measured as age accelerated residual, between opioid-dependent subjects and opioid-exposed controls.

**Supplementary Figure 7. Power analysis.**

At a genome-wide level and assuming a moderate effect size (d=0.5-0.8), a sample size of 218 has 60% power in a case-control design. Power was computed using G*Power 3.1 in a Wilcoxon-Mann-Whitney two-group test.

**Supplementary Figure 1. Density plots.**

**
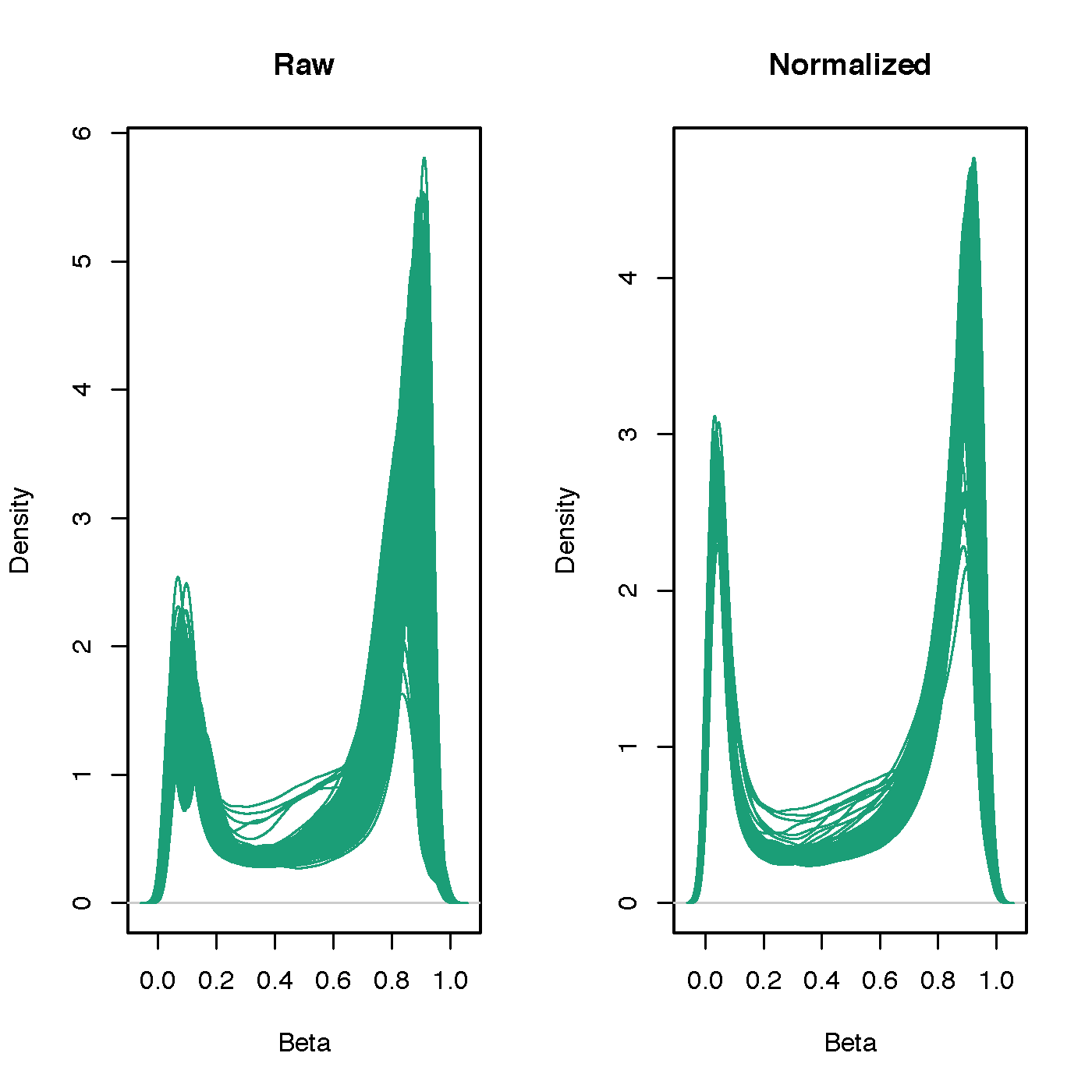
**

**Supplementary Figure 2. Quantile-quantile plot.**

**
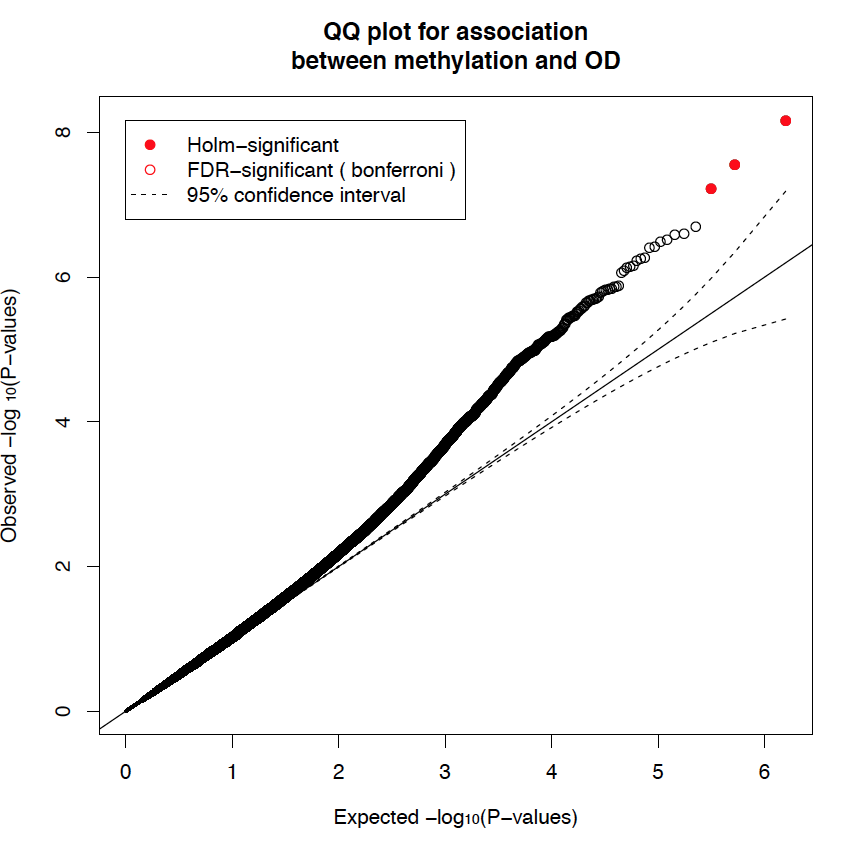
**

**Supplementary Figure 3. Brain-blood methylation patterns of GWS CpG sites.**

**
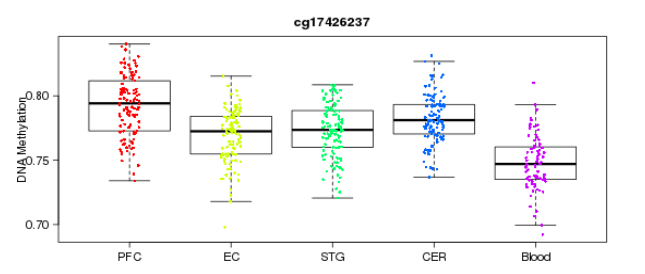
**

**Supplementary Figure 4. Brain-blood gene expression patterns of genes from GWS CpG sites.**

**A.**

**
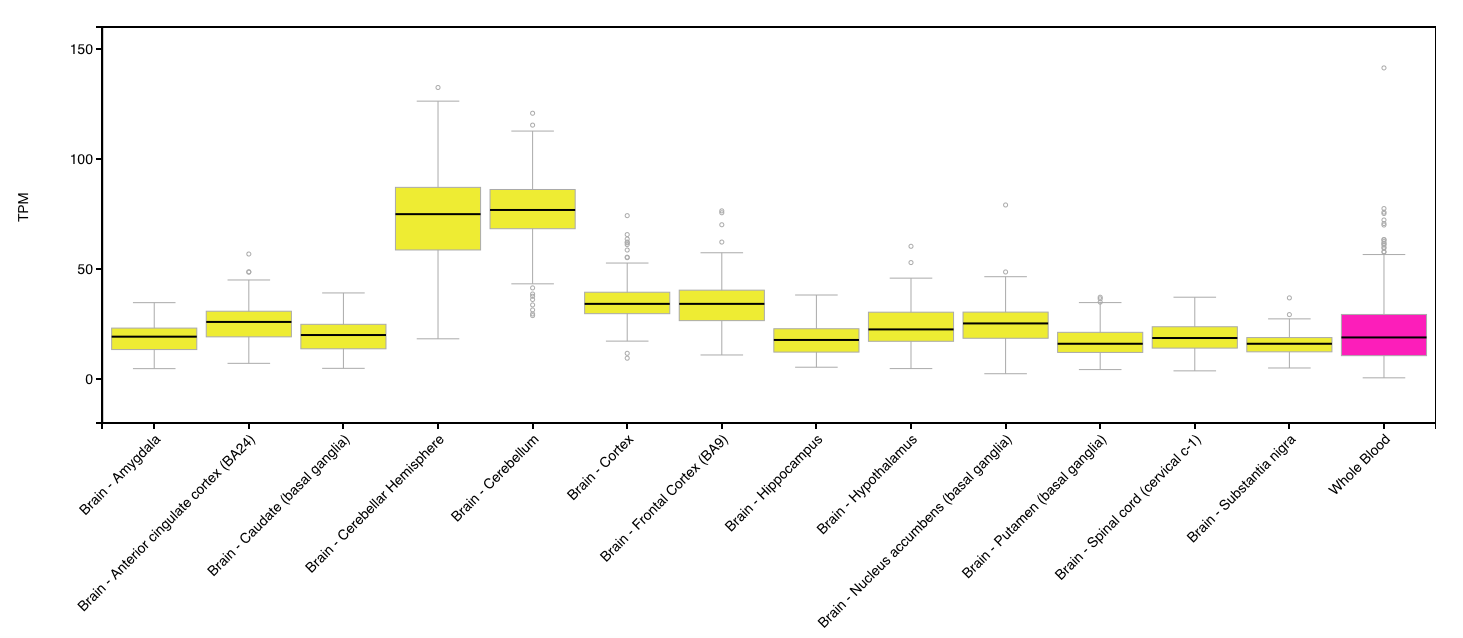
**

**B.**

**
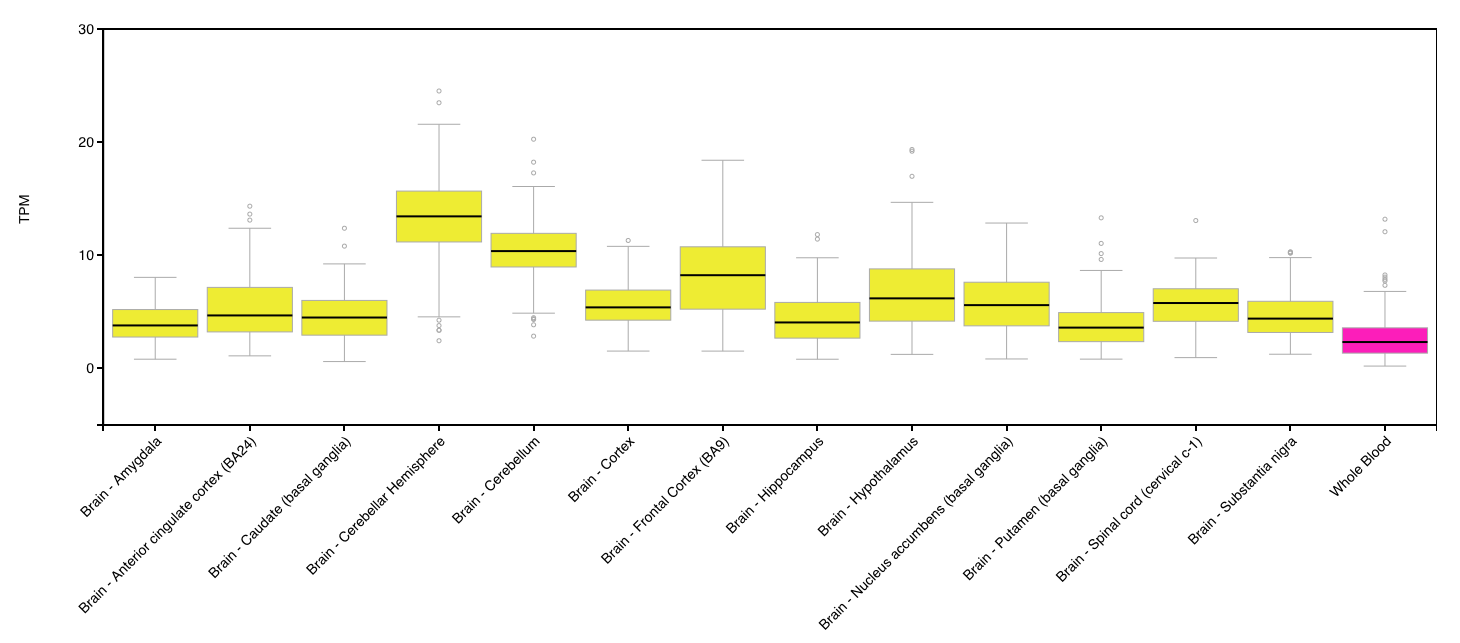
**

**Supplementary Figure 5. Association between genome-wide significant (GWS) CpG sites and other clinical variables.**

**C)**

**B)**

**A)**

**Current smoking**

**Cocaine dependence**

**Alcohol dependence**

**cg17426237**

**D)**

**E)**

**F)**

**cg21381136**

**I)**

**H)**

**G)**

**cg18177613**

**Supplementary Figure 6. Epigenetic age acceleration in peripheral blood of opioid-dependent subjects and opioid-exposed controls.**

**A.**

**
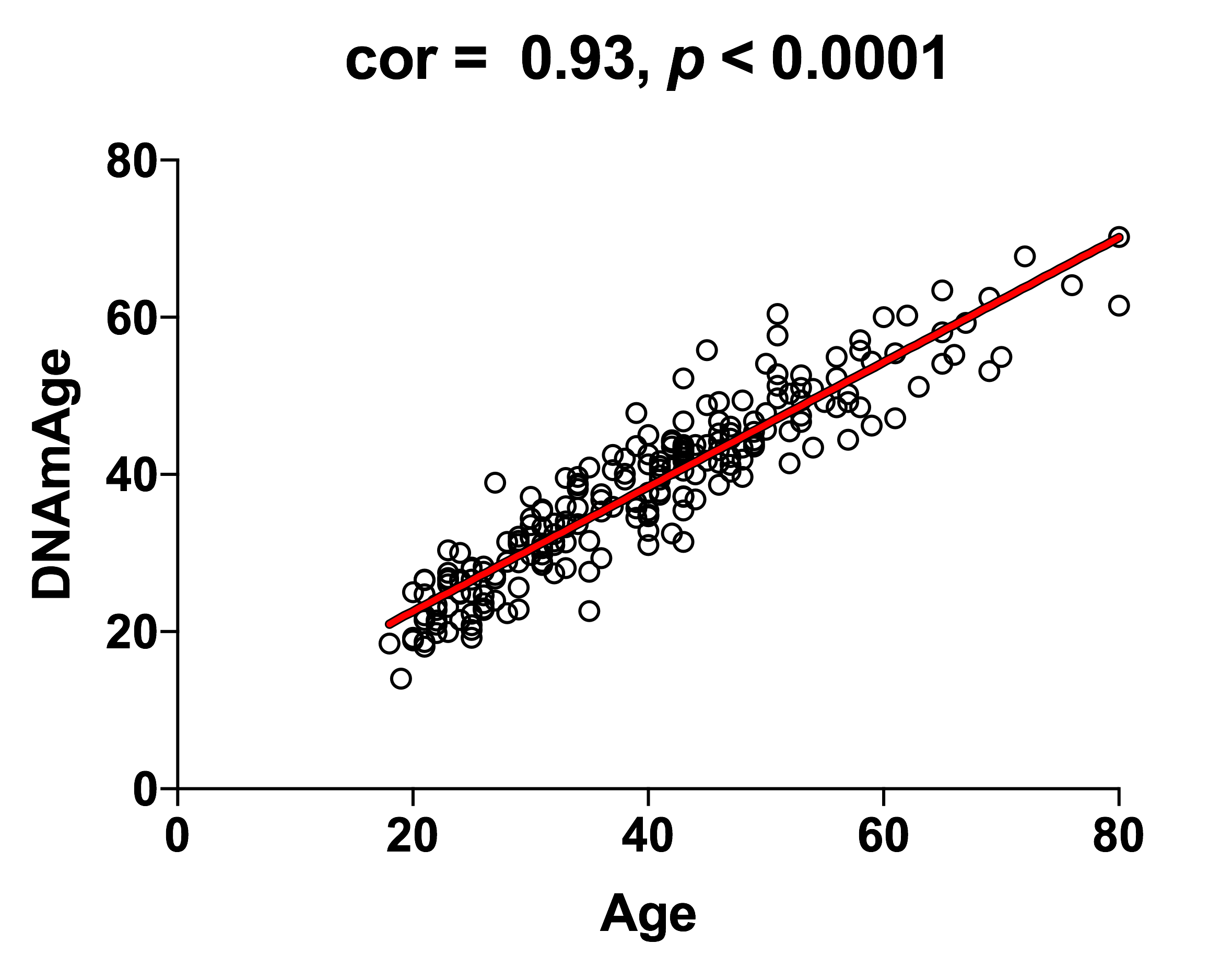
**

**B.**

**
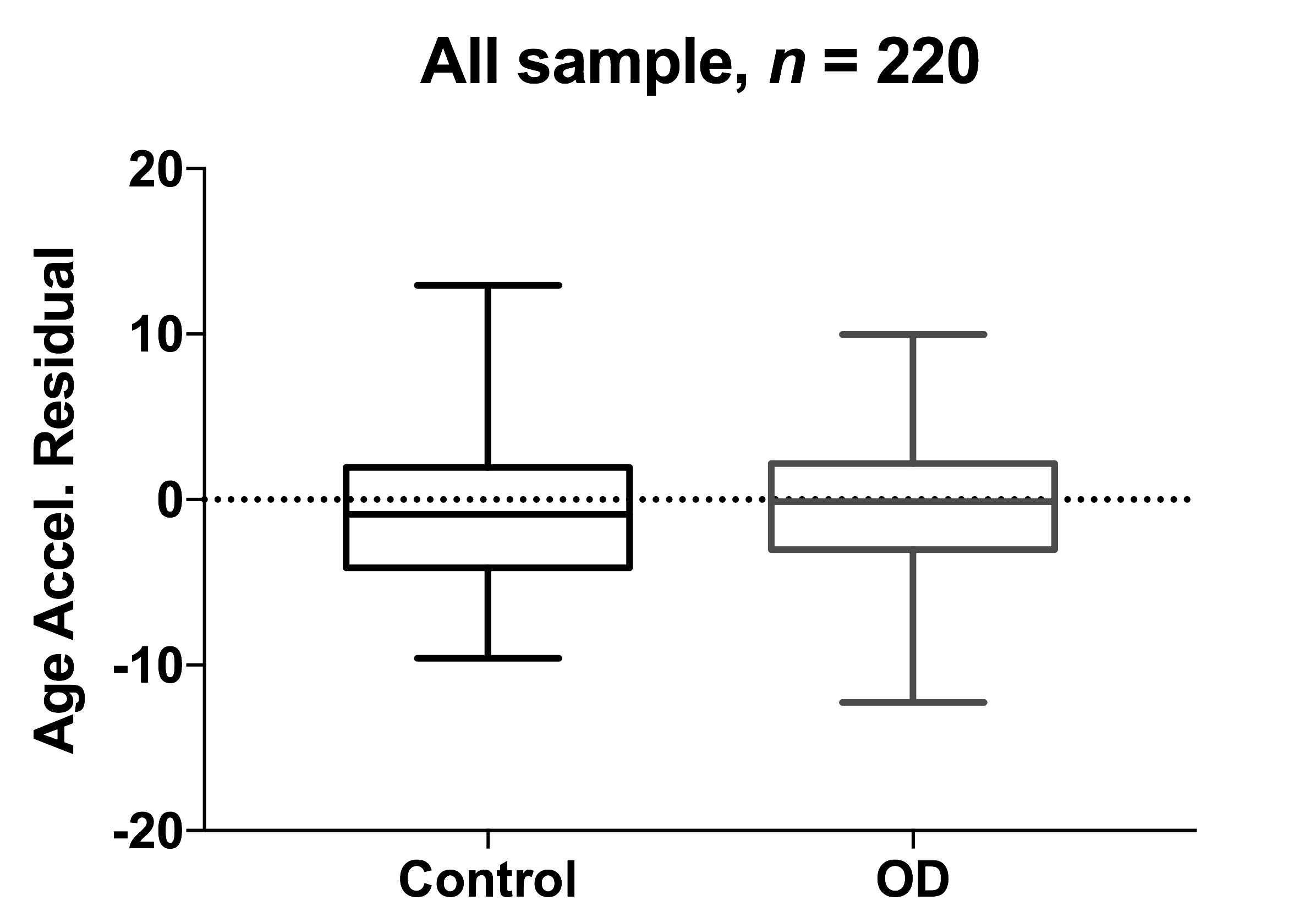
**

**Supplementary Figure 7. Power analysis.**

**References:**

1. Faul F, Erdfelder E, Lang AG, Buchner A. G*Power 3: a flexible statistical power analysis program for the social, behavioral, and biomedical sciences. *Behavior research methods.* 2007;39(2):175-191.

2. Tsai PC, Bell JT. Power and sample size estimation for epigenome-wide association scans to detect differential DNA methylation. *Int J Epidemiol.* 2015;44(4):1429-1441.

3. Weder N, Zhang H, Jensen K, Yang BZ, Simen A, Jackowski A, et al. Child abuse, depression, and methylation in genes involved with stress, neural plasticity, and brain circuitry. *Journal of the American Academy of Child and Adolescent Psychiatry.* 2014;53(4):417-424 e415.

4. Hannon E, Lunnon K, Schalkwyk L, Mill J. Interindividual methylomic variation across blood, cortex, and cerebellum: implications for epigenetic studies of neurological and neuropsychiatric phenotypes. *Epigenetics.* 2015;10(11):1024-1032.

5. Horvath S. DNA methylation age of human tissues and cell types. *Genome biology.* 2013;14(10):R115.
